# Adaptive Nonlinear Bernstein-Guided Parrot Optimizer for Mural Image Segmentation

**DOI:** 10.3390/biomimetics10080482

**Published:** 2025-07-22

**Authors:** Jianfeng Wang, Jiawei Fan, Xiaoyan Zhang, Bao Qian

**Affiliations:** 1 College of Design, Hanyang University, Ansan 15588, Republic of Korea; 15306435766@163.com (J.W.); zxy20241@hanyang.ac.kr (X.Z.); baoqian@hanyang.ac.kr (B.Q.); 2 School of Physics and Electronic Engineering, Xinjiang Normal University, Urumqi 830054, China

**Keywords:** parrot optimizer, adaptive learning strategy, nonlinear factor, third-order Bernstein-guided strategy, mural image segmentation

## Abstract

During the long-term preservation of murals, the degradation of mural image information poses significant challenges to the restoration and conservation of world cultural heritage. Currently, mural conservation scholars focus on image segmentation techniques for mural restoration and protection. However, existing image segmentation methods suffer from suboptimal segmentation quality. To improve mural image segmentation, this study proposes an efficient mural image segmentation method termed Adaptive Nonlinear Bernstein-guided Parrot Optimizer (ANBPO) by integrating an adaptive learning strategy, a nonlinear factor, and a third-order Bernstein-guided strategy into the Parrot Optimizer (PO). In ANBPO, First, to address PO’s limited global exploration capability, the adaptive learning strategy is introduced. By considering individual information disparities and learning behaviors, this strategy effectively enhances the algorithm’s global exploration, enabling a thorough search of the solution space. Second, to mitigate the imbalance between PO’s global exploration and local exploitation phases, the nonlinear factor is proposed. Leveraging its adaptability and nonlinear curve characteristics, this factor improves the algorithm’s ability to escape local optimal segmentation thresholds. Finally, to overcome PO’s inadequate local exploitation capability, the third-order Bernstein-guided strategy is introduced. By incorporating the weighted properties of third-order Bernstein polynomials, this strategy comprehensively evaluates individuals with diverse characteristics, thereby enhancing the precision of mural image segmentation. ANBPO was applied to segment twelve mural images. The results demonstrate that, compared to competing algorithms, ANBPO achieves a 91.6% win rate in fitness function values while outperforming them by 67.6%, 69.4%, and 69.7% in PSNR, SSIM, and FSIM metrics, respectively. These results confirm that the ANBPO algorithm can effectively segment mural images while preserving the original feature information. Thus, it can be regarded as an efficient mural image segmentation algorithm.

## 1. Introduction

Murals, as a vital component of world cultural heritage, embody profound cultural and historical value [1]. However, during prolonged preservation, the information within mural images degrades, posing significant challenges to the restoration and conservation of world cultural heritage [2]. Currently, scholars in mural conservation are dedicated to restoring and preserving murals through extensive scientific approaches, aiming to safeguard their historical, artistic, and scientific significance [3]. Within the restoration process of mural images, locating degraded details is of critical importance [4]. For instance, Hou et al. utilized hyperspectral imaging to extract hidden information from murals, thereby enhancing the visual value of ancient mural patterns [5]. Wu et al. integrated a generative adversarial network for digital restoration of murals, contributing to the advancement of mural restoration techniques [6]. Xiao et al. proposed a multi-level residual network for restoring edges and contours in ancient murals, with the core idea of leveraging image segmentation across different frequency domains [7]. Zhou et al. combined a progressive context refinement network to restore texture and structural features in ancient mural images [8]. Yu et al. introduced a mural extraction method based on image enhancement and edge detection to capture edge information from murals, ensuring high-quality image restoration [9]. These existing studies primarily focus on restoring mural images. However, due to insufficient data support, neural network methods gradually have limitations. Among which, segmenting mural images also serves as a means to extract critical feature information. Image segmentation technology serves as a key technique for identifying image details by partitioning an image into multiple regions based on its feature information, thereby facilitating the recognition of detailed image elements [10]. Among current methods, metaheuristic-based image segmentation approaches have garnered substantial attention due to their simple structure, low computational complexity, and broad applicability [11].

Metaheuristic algorithms represent a class of algorithms characterized by their simplicity and computational efficiency, primarily developed by simulating biological behaviors and natural phenomena observed in the real world. Common categorizations divide metaheuristic algorithms into four primary types: evolution-based, swarm-based, physics/chemistry-inspired, and human-based [12]. Evolution-based metaheuristic algorithms include Differential Evolution (DE) [13], Genetic Algorithm (GA) [14], and Biogeography-Based Optimization (BBO) [15]. Swarm-based metaheuristic algorithms include Particle Swarm Optimization (PSO) [16], Slime Mold Algorithm (SMA) [17], and Whale Optimization Algorithm (WOA) [18]. Physics/chemistry-inspired metaheuristic algorithms include Multi-verse Optimizer (MVO) [19], Atom Search Optimization (ASO) [20], and Water Evaporation Optimization (WEO) [21]. Human-based metaheuristic algorithms include Teaching–Learning-Based Optimization (TLBO) [22], Poor and Rich Optimization (PRO) [23], and Search And Rescue Optimization (SAR) [24]. Given their computational efficiency and ease of implementation, researchers have proposed numerous metaheuristic-based image segmentation algorithms to enhance the quality of image segmentation.

In recent years, researchers have introduced a variety of metaheuristic-based image segmentation algorithms to address challenges in medical and complex image analysis. For instance, Houssein et al. proposed the Snake Optimization algorithm with Opposition-Based Learning (SO-OBL) to improve CT scan segmentation for liver disease diagnostics. Experimental results revealed that SO-OBL excels in global optimization and multi-level segmentation, outperforming competing metaheuristic algorithms across FSIM, SSIM, and PSNR metrics, thereby validating its efficiency in computer-aided diagnostic systems [25]. Wang et al. proposed an enhanced Northern Goshawk Optimization Algorithm combining three learning strategies to solve the mural image segmentation problem, achieving excellent results on eight standard images [26]. Qiao et al. developed a hybrid Arithmetic Optimization Algorithm and Harris Hawks Optimizer (AOA-HHO) for multi-level threshold segmentation. By evaluating seven threshold levels, AOA-HHO achieved superior performance in segmentation accuracy, fitness function values, PSNR, and SSIM, outperforming baseline algorithms [27]. Yuan et al. proposed the Artemisinin Optimization (AO) Algorithm to tackle medical image segmentation challenges. By balancing global exploration and local exploitation, AO demonstrated strong optimization performance across six-threshold segmentations of 15 breast cancer pathology images, surpassing eight high-performing algorithms in accuracy, feature similarity, PSNR, and SSIM [28]. Chen et al. addressed segmentation inefficiencies with the Poplar Optimization Algorithm (POA), which enhances population diversity to improve optimization. Experiments on six standard images confirmed POA’s superior multi-threshold segmentation performance [29]. Wang et al. mitigated the Whale Optimization Algorithm’s (WOA) limitations such as weak local search and premature convergence by introducing WOA with Crossover and Removal of Similarity (CRWOA). In multi-level thresholding of 10 grayscale images, CRWOA outperformed five comparison algorithms in convergence speed and segmentation quality [30]. Arunita et al. combined the Lévy-Cauchy Arithmetic Optimization Algorithm (LCAOA) with Rough K-Means (RKM) to balance exploration and exploitation via Lévy flight and Cauchy distribution, while opposition-based learning enhanced efficiency. Experiments on diverse image types (e.g., color, oral pathology, and leaf images) showed high feature similarity and accuracy [31]. Wang et al. proposed a multi-threshold segmentation method for breast cancer images using an improved Dandelion Optimization Algorithm. By integrating opposition-based learning, the method achieved more precise lesion segmentation and superior performance metrics, validating its effectiveness in handling complex cellular structures [32].

Previous studies have validated the effectiveness of metaheuristic-based image segmentation methods and demonstrated that integrating learning strategies enhances their segmentation performance. However, existing metaheuristic approaches exhibit limitations when addressing domain-specific segmentation challenges, such as mural image segmentation, where they often converge to suboptimal threshold combinations, resulting in poor retention of feature information and degraded segmentation quality. Motivated by these challenges, there is an urgent need to propose a novel optimization algorithm with efficient segmentation capabilities for mural image segmentation. Fortunately, the Parrot Optimizer (PO) has been proven as an algorithm with robust optimization performance, demonstrating strong scalability and adaptability across various optimization problems [33]. Consequently, this study selects PO for mural image segmentation. However, as mural image complexity increases, the original PO faces inevitable shortcomings, including insufficient global exploration, inadequate local exploitation, and an imbalance between exploration and exploitation phases, which may still lead to suboptimal segmentation performance. To address these issues, this study introduces an enhanced PO algorithm termed ANBPO by integrating three learning strategies: First, an adaptive learning strategy is proposed to overcome PO’s limited global exploration capability. By considering individual information disparities and learning behaviors, this strategy effectively enhances global exploration, enabling the algorithm to thoroughly search the solution space and improve mural image segmentation quality. Second, a nonlinear factor is introduced to balance exploration and exploitation phases. Leveraging its adaptability and nonlinear curve characteristics, this factor mitigates the imbalance, enabling the algorithm to escape local optimal threshold combinations and enhance mural image segmentation quality. Finally, a third-order Bernstein-guided strategy is proposed to improve PO’s local exploitation capability. By incorporating the weighted properties of third-order Bernstein polynomials, this strategy comprehensively evaluates individuals with diverse characteristics, thereby strengthening local exploitation and improving mural image segmentation precision. The primary contributions of this study are as follows:

- The adaptive learning strategy is proposed to enhance the algorithm’s global exploration capability by accounting for individual information disparities and learning behaviors.
- The nonlinear factor is introduced to balance the algorithm’s exploration and exploitation phases, leveraging its adaptability and nonlinear curve characteristics.
- The third-order Bernstein-guided strategy is proposed to strengthen the algorithm’s local exploitation capability by incorporating the weighted properties of third-order Bernstein polynomials, enabling comprehensive evaluation of individuals with diverse characteristics.
- By integrating these three learning strategies into the PO, an enhanced PO algorithm termed ANBPO is developed.
- Experimental segmentation of twelve mural images using ANBPO confirms its potential as a promising algorithm for mural image segmentation.

The subsequent work plan of this study is outlined as follows: Section 2 introduces the mathematical model and execution logic of the PO. Section 3 proposes the ANBPO algorithm by integrating three learning strategies into PO. Section 4 applies ANBPO to solve the mural image segmentation problem using twelve mural images to evaluate the algorithm’s performance. Section 5 presents the study conclusions and outlines future work directions.

## 2. The Mathematical Model of the Parrot Optimizer

This section primarily introduces the concept of the PO. PO achieves global exploration and local exploitation capabilities of the algorithm primarily by simulating the foraging behavior, staying behavior, communicating behavior, and fear of strangers’ behavior of parrots in nature, thereby forming an optimization algorithm with excellent performance. Its mathematical model mainly comprises five components: the population initialization phase, foraging behavior, staying behavior, communicating behavior, and fear of strangers’ behavior. Below, the mathematical models of these five parts will be described, and the execution logic of PO will be subsequently introduced. It should be noted that the mathematical models and formulas in this section are sourced from literature on the original NGO algorithm [33].

### 2.1. Population Initialization Phase

The initialization phase of the PO is designed to generate an initial population for use in the algorithm’s iterative process. Each individual within the population represents a candidate solution to the optimization problem being solved. Every individual is generated within the upper and lower bounds of the variables in the optimization problem, as expressed in Equation (1).

(1) $$X_{i}^{0}=lb+rand(0,1)\cdot (ub-lb),\;i=1,2,\ldots ,N$$

where, $X_{i}^{0}$ represents the information of the ith individual in the initial population. lb and ub denote the lower and upper bounds of the optimization problem, respectively, and are represented as 1×dim vectors, where dim signifies the dimensionality of the variables in the optimization problem. rand(0,1) indicates a random number generated within the interval [0, 1], and N represents the size of the population. After generating an initial population of size N, solution refinement is conducted by simulating the parrot’s foraging behavior, staying behavior, communicating behavior, and fear of strangers’ behavior.

### 2.2. Foraging Behavior

This section primarily focuses on mathematical modeling of the parrot’s foraging behavior. In this behavior, the parrot primarily moves based on the location of food sources, and its positional update is described by Equation (2).

(2) $$X_{i}^{t+1}=\left(X_{i}^{t}-X_{best}\right)\cdot Levy(dim)+rand\cdot {\left(1-\frac{t}{Max_{iter}}\right)}^{\frac{2t}{Max_{iter}}}\cdot X_{mean}^{t}$$

where, $X_{i}^{t+1}$ represents the information of the ith individual at the (t+1)th iteration, $X_{i}^{t}$ denotes the information of the ith individual at the tth iteration, $X_{best}$ signifies the optimal individual within the population, $X_{best}$ is defined as the threshold value such that, when utilized to segment the mural image, the resulting segmented mural image exhibits the maximum inter-class variance with respect to the original image. Consequently, $X_{best}$ is considered the optimal individual within the population, representing the optimal combination of segmentation thresholds. Levy(dim) indicates a random number generated from a Lévy distribution with a parameter dim, rand represents a random number generated within the interval [0, 1], $Max_{iter}$ denotes the maximum number of iterations, and $X_{mean}^{t}$ represents the average information of the individuals in the population at the tth iteration, as expressed by Equation (3).

(3) $$X_{mean}^{t}=\frac{1}{N}\sum_{i=1}^{N}X_{i}^{t}$$

### 2.3. Staying Behavior

This section primarily focuses on mathematical modeling of the parrot’s staying behavior, where the positional update is described by Equation (4).

(4) $$X_{i}^{t+1}=X_{i}^{t}+X_{best}\cdot Levy(dim)+rand\cdot ones(1,dim)$$

where, ones(1,dim) represents the generation of a vector of all ones with a size of 1×dim.

### 2.4. Communicating Behavior

This section primarily focuses on mathematical modeling of the parrot’s communicating behavior. The parrot’s communicating behavior mainly involves two approaches: interacting with the group and not interacting with the group. Here, it is assumed that both communication methods occur with equal probability. The parrot’s communicating behavior is described by Equation (5).

(5) $$X_{i}^{t+1}=\left\{\begin{array}{l} 0.2\cdot rand\cdot (1-\frac{t}{Max_{iter}})\cdot (X_{i}^{t}-X_{mean}^{t}),\;P\leq 0.5\\ 0.2\cdot rand\cdot exp\left(-\frac{t}{rand\cdot Max_{iter}}\right),\;\;\;P>0.5 \end{array}\right.$$

where, exp(⋅) denotes the exponential operation, and P represents a random number generated within the interval [0, 1].

### 2.5. Fear of Strangers’ Behavior

This section primarily focuses on mathematical modeling of the parrot’s fear of strangers’ behavior. In this behavior, the parrot exhibits fear toward strangers and attempts to move closer to the optimal individual within the population to seek a safer environment. Its positional update is represented by Equation (6).

(6) $$\begin{array}{c} X_{i}^{t+1}=X_{i}^{t}+rand\cdot cos(0.5\pi \cdot \frac{t}{Max_{iter}})\cdot (X_{best}-X_{i}^{t})\\ -cos(rand\cdot \pi )\cdot {\left(\frac{t}{Max_{iter}}\right)}^{\frac{2}{Max_{iter}}}\cdot (X_{i}^{t}-X_{best}) \end{array}$$

where, cos(⋅) denotes the cosine function operation.

### 2.6. Implementation of Parrot Optimizer

When solving practical optimization problems, the PO first generates an initial population with a certain level of optimization capability. Subsequently, it conducts global exploration and local exploitation of the initial solutions by simulating the parrot’s foraging behavior, staying behavior, communicating behavior, and fear of strangers’ behavior. This approach enhances the quality of the solutions, thereby improving the resolution of the optimization problem. Algorithm 1 presents the pseudocode for the PO.

**Algorithm 1:** Pseudo-code of PO
1: Initialization: N, ub, lb, dim$,\;Max_{iter}$2: Generate initialized population using Equation (1)3: ***for***$\;i=1:Max_{iter}$ ***do***4:   Calculate fitness function value5:   Save the best individual6:   ***for*** j=1:N ***do***7:    St=randi([1,4])8:    Foraging behavior9:    ***if*** St==1 ***Then***10:     Updating the individual using Equation (2)11:    Staying behavior12:    ***else if*** St==2 ***Then***13:     Updating the individual using Equation (4)14:    Communicating behavior15:    ***else if*** St==3 ***Then***16:     Updating the individual using Equation (5)17:    Fear of strangers’ behavior18:    ***else if*** St==4 ***Then***19:     Updating the individual using Equation (6)20:    ***end*** ***if***21:   ***end*** ***for***22:  ***end for***23: Return the best individual


## 3. The Mathematical Model of the ANBPO

The original PO exhibits deficiencies when solving mural image segmentation problems, including insufficient global exploration capability, inadequate local exploitation capability, and an imbalance between the exploration and exploitation phases. These shortcomings often cause the algorithm to converge prematurely to locally optimal segmentation thresholds, thereby degrading the quality of the segmented images. To mitigate these limitations, this section proposes an enhanced variant of PO, termed ANBPO, by integrating an adaptive learning strategy, a nonlinear factor, and a third-order Bernstein-guided strategy. First, to address the algorithm’s inadequate global exploration capability, an adaptive learning strategy is introduced. By accounting for individual differences in information and learning adaptability, this strategy effectively broadens the search space, enabling the algorithm to explore solutions more comprehensively and improve the quality of mural image segmentation. Second, to resolve the imbalance between global exploration and local exploitation, a nonlinear factor is proposed. Leveraging its adaptive nature and nonlinear curve characteristics, this factor dynamically balances the two phases, enhancing the algorithm’s ability to escape local optima and improving the segmentation quality of mural images. Finally, to strengthen the algorithm’s local exploitation capability, a third-order Bernstein-guided strategy is introduced. By incorporating the weighted properties of the third-order Bernstein polynomial, this strategy comprehensively evaluates individuals with diverse characteristics, thereby refining local exploitation and increasing the precision of mural image segmentation. The subsequent sections will detail the implementations of the adaptive learning strategy, the nonlinear factor, and the third-order Bernstein-guided strategy.

### 3.1. Adaptive Learning Strategy

**Figure 1:**
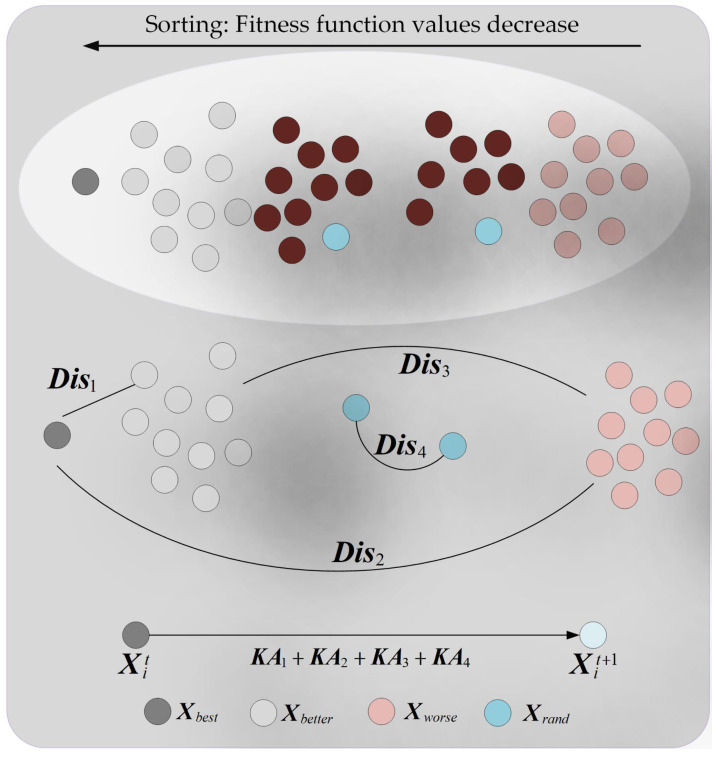
Schematic of adaptive learning strategy.

The original PO algorithm suffers from insufficient global exploration capability when solving mural image segmentation problems, which hinders its ability to effectively search the solution space. To address this issue, this section proposes an adaptive learning strategy to enhance PO’s global exploration capability; the schematic diagram of the adaptive learning strategy is shown in Figure 1. The core idea of the adaptive learning strategy is that individuals learn from different types of disparity information while adaptively accounting for their own learning adaptability and the learnability of varying disparity information. This approach significantly improves the algorithm’s global exploration capability, leading to higher-quality segmentation of mural images. Specifically, the adaptive learning strategy considers four groups of disparities: (1) the disparity between the best individual and better individuals ($Dis_{1}$), (2) the disparity between the best individual and worse individuals ($Dis_{2}$), (3) the disparity between better individuals and worse individuals ($Dis_{3}$), and (4) the disparity between two randomly selected individuals ($Dis_{4}$). These four groups of disparities are represented using Equation (7).

(7) $$\left\{\begin{array}{l} Dis_{1}=X_{best}-X_{better}\\ Dis_{2}=X_{best}-X_{worse}\\ Dis_{3}=X_{better}-X_{worse}\\ Dis_{4}=X_{rand1}-X_{rand2} \end{array}\right.$$

where, $X_{best}$ denotes the best individual in the population. $X_{better}$ represents a randomly selected individual from the set of the top 10 individuals with the smallest fitness function values in the population. $X_{worse}$ denotes a randomly selected individual from the set of the top 10 individuals with the largest fitness function values in the population. $X_{rand1}$ and $X_{rand2}$ represent two distinct randomly selected individuals from the population. In this section, considering the varying learnability of each group of information disparities, the learnability of each group is represented using Equation (8).

(8) $$LF_{k}=\frac{\left\|Dis_{k}\right\|}{\sum_{k=1}^{4}\left\|Dis_{k}\right\|},(k=1,2,3,4)$$

where, $LF_{k}$ denotes the learnability of the kth group of information disparities, and “‖ ‖” represents the modulus operation. In the adaptive learning strategy, individuals possess varying learning capabilities. Considering that higher-quality individuals should reduce their learning intensity while lower-quality individuals should increase theirs, the learning capability of each individual is defined using Equation (9).

(9) $$SF_{i}=\frac{fit_{i}}{fit_{max}},\;(1\leq i\leq N)$$

where, $SF_{i}$ denotes the learning capability of the ith individual, $fit_{i}$ represents the fitness function value of the ith individual, where, the fitness function value $fit_{i}$ is defined as follows: First, the individual $X_{i}$ is employed as the threshold combination for segmenting the mural image. Subsequently, the mural image is segmented using this threshold combination. Then, the inter-class variance between the original image and the segmented image is calculated, and this calculated inter-class variance value is defined as the fitness function value. and $fit_{max}$ indicates the maximum fitness function value among individuals in the population. Subsequently, the learning process of the ith individual from the kth group of information disparities is represented using Equation (10).

(10) $$KA_{k}=arctan(2\cdot \pi \cdot (1-\frac{t}{Max_{iter}}))\cdot SF_{i}\cdot LF_{k}\cdot Dis_{k},(k=1,2,3,4)$$

where, $KA_{k}$ denotes the amount of information acquired by the ith individual through learning from the kth group of disparities, and arctan represents the arctangent function. Based on Equation (10), the new state generated by the ith individual after learning from the four groups of information disparities is expressed as Equation (11).

(11) $$X_{i}^{t+1}=X_{i}^{t}+KA_{1}+KA_{2}+KA_{3}+KA_{4}$$

Subsequently, the individual’s state is preserved using Equation (12).

(12) $$X_{i}^{t+1}=\left\{\begin{array}{l} X_{i}^{t+1}\;if\;fit_{i}^{t+1}<fit_{i}\\ X_{i}^{t}\;\;otherwise \end{array}\right.$$

where, $fit_{i}^{t+1}$ represents the fitness function value of the individual $X_{i}^{t+1}$. By adaptive learning to different information disparities through an adaptive learning strategy, the algorithm’s global exploration capability can be effectively enhanced, ensuring thorough exploration of the solution space and thereby improving the quality of mural image segmentation.

### 3.2. Nonlinear Factor

**Figure 2:**
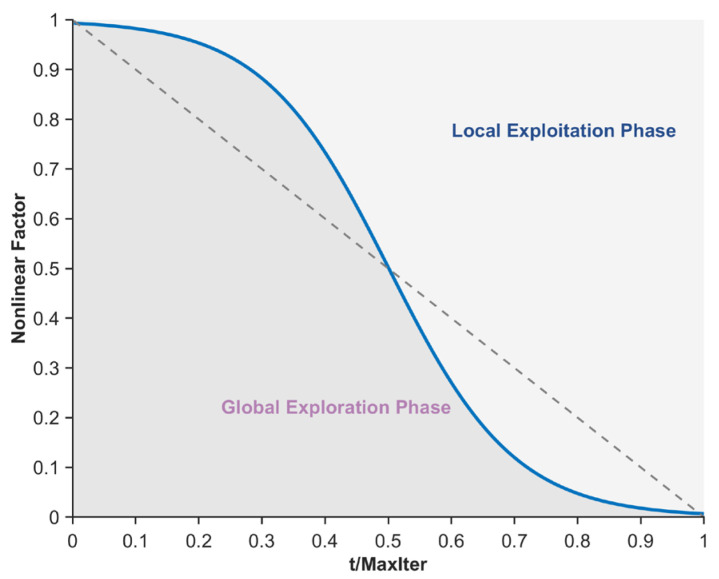
Sigmod function term.

**Figure 3:**
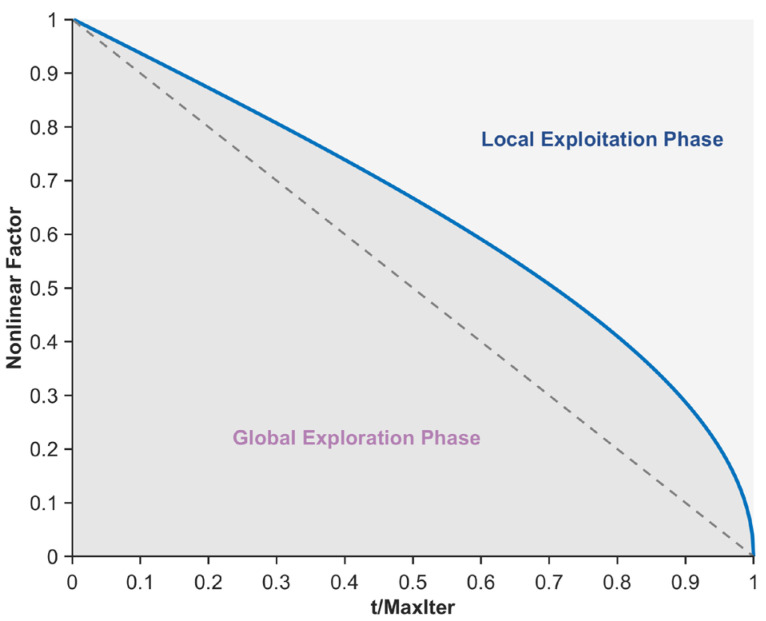
Inverse cosine function term.

**Figure 4:**
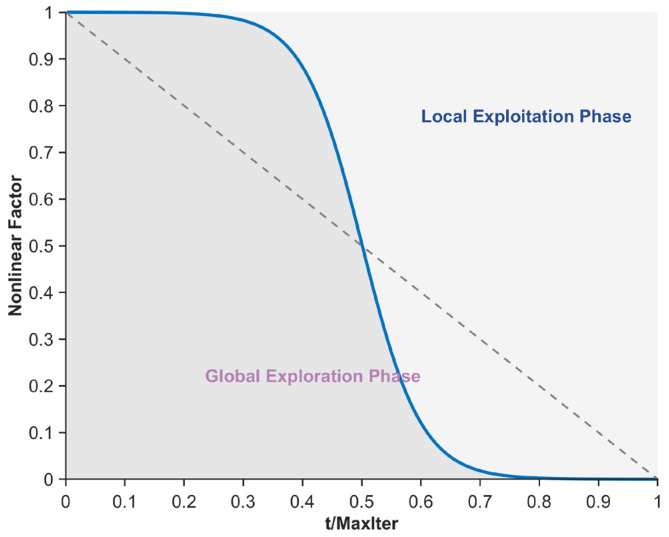
Arctangent function term.

**Figure 5:**
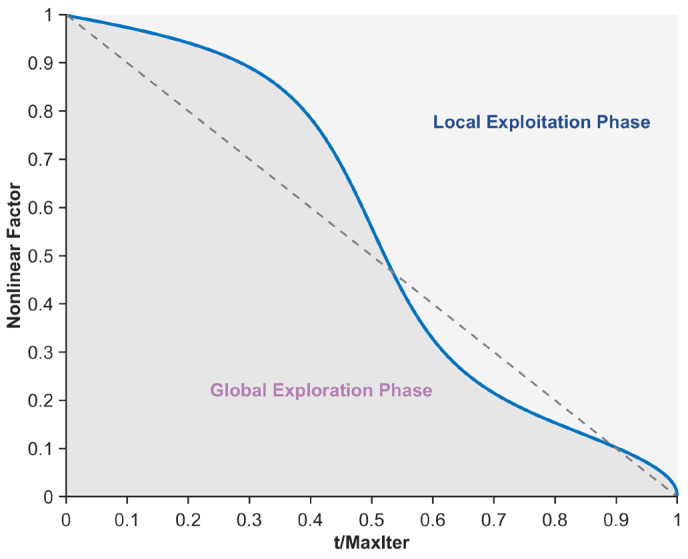
Nonlinear factor.

The original PO exhibits an imbalance between the global exploration and local exploitation phases when solving the mural image segmentation problem, leading to a tendency to fall into local suboptimal segmentation threshold traps, thereby compromising the quality of mural image segmentation. To mitigate this issue, this section proposes a nonlinear factor to balance the global exploration and local exploitation phases. By integrating the Sigmoid function, arccosine function, and arctangent function, the balancing capability of the nonlinear factor is enhanced, enabling the algorithm to exhibit better global exploration performance in the early iterations. As iterations progress, local exploitation gradually dominates while retaining a certain level of global exploration capability. This phenomenon effectively reduces the likelihood of the algorithm getting trapped in local optima during mural image segmentation. Specifically, the nonlinear factor comprises three terms: a Sigmoid function term, an arccosine function term, and an arctangent function term. Among these, the Sigmoid function term primarily controls the balance during the mid-iteration phase and is expressed using Equation (13).

(13) $$NF_{sigmod}=1-\frac{1}{1+exp(-k\cdot (t/Max_{iter}-0.5))}$$

where, $NF_{sigmod}$ denotes the Sigmoid function term and exp(⋅) represents the exponential operation. In this study, the parameter k is set to 10. The curve depicting the variation of $NF_{sigmod}$ with the number of iterations is shown in Figure 2. As illustrated in the figure, the Sigmoid function curve approaches equilibrium during the mid-iteration phase, effectively ensuring a balance between the global exploration and local exploitation phases of the algorithm, thereby guaranteeing a certain level of quality in mural image segmentation. However, its limitations include insufficient exploitation capability in the early iterations and inadequate exploration capability in the late iterations, indicating certain drawbacks. To address the deficiency in global exploration capability during the late iterations caused by the Sigmoid function term, an arccosine function term is subsequently introduced to sustain the algorithm’s global exploration capability in the late iterations, as expressed in Equation (14).

(14) $$NF_{arccos}=arccos(\frac{t}{Max_{iter}})/(\frac{\pi }{2})$$

where, $NF_{arccos}$ denotes the arccosine function term and arccos(⋅) represents the arccosine operation. The curve depicting the variation of $NF_{arccos}$ with the number of iterations is shown in Figure 3. As illustrated in the figure, the arccosine function term provides a greater proportion of global exploration, thereby enhancing the algorithm’s global exploration capability during the late iterations. Additionally, to address the deficiency in local exploitation capability during the early iterations caused by the Sigmoid function term, an arctangent function term is subsequently introduced to ensure the algorithm’s local exploitation capability in the early iterations, as expressed in Equation (15).

(15) $$NF_{arctan}=(arctan(5-10\cdot (\frac{t}{Max_{iter}}))+1)/2$$

where, $NF_{arctan}$ denotes the arctangent function term, and arctan(⋅) represents the arctangent operation. The curve depicting the variation of $NF_{arctan}$ with the number of iterations is shown in Figure 4. As illustrated in the figure, the arctangent function term provides a greater proportion of local exploitation during the early iterations, thereby enhancing the algorithm’s local exploitation capability in this phase. In summary, by integrating the properties of the Sigmoid function, arccosine function, and arctangent function, the nonlinear factor proposed in this section is formulated to achieve a more reasonable balance between the algorithm’s global search and local exploitation phases, as expressed in Equation (16).

(16) $$NF=\frac{\left(NF_{sigmod}+NF_{arccos}+NF_{arctan}\right)}{3}$$

where, NF denotes the nonlinear factor proposed in this section, and its variation curve with respect to the number of iterations is shown in Figure 5. As illustrated in the figure, during the early iterations, the algorithm’s global exploration phase dominates while still retaining a certain level of local exploitation capability. As iterations progress, during the mid-iteration phase, the algorithm’s global exploration and local exploitation phases approach equilibrium. In the late iterations, the algorithm’s local exploitation capability becomes dominant, yet it maintains a relatively high level of global exploration capability, thereby strengthening its ability to escape local optimal segmentation thresholds. In summary, the nonlinear factor proposed in this section achieves a more reasonable balance between the exploration and exploitation phases of the algorithm, effectively enhancing the quality of mural image segmentation.

### 3.3. Third-Order Bernstein-Guided Strategy

The original PO exhibits a deficiency in local exploitation capability when solving the mural image segmentation problem, leading to a loss in segmentation accuracy and an inability to effectively preserve meaningful image information. Zhang et al. [34] pointed out that individuals can effectively enhance the algorithm’s local exploitation by learning from those with superior performance. Additionally, Wu et al. [35] indicated that incorporating Bernstein polynomials to guide individuals can further improve the algorithm’s local exploitation capability. Inspired by these insights, to further enhance the local exploitation of the PO algorithm and achieve higher optimization precision, this section proposes a third-order Bernstein-guided strategy. By leveraging the property that the weights of the third-order Bernstein polynomial sum to 1, individuals with distinct characteristics are weighted to form weighted individuals. These weighted individuals are then used to guide the population, effectively strengthening the algorithm’s local exploitation capability and improving the quality of mural image segmentation. First, the n-order Bernstein polynomial is expressed using Equation (17).

(17) $$B_{w,n}(p)=C_{n}^{w}\cdot p^{w}\cdot {\left(1-p\right)}^{n-w}$$

where, 0≤p≤1 denotes the probability of an event occurring, w represents the number of successful occurrences of the event, n indicates the total number of independent and non-repetitive trials, and $C_{n}^{w}$ is expressed using Equation (18).

(18) $$C_{n}^{w}=\frac{n!}{w!(n-w)!}$$

where, ‘!’ denotes the factorial operation. For a third-order Bernstein polynomial, n=3, and w=0,1,2,3, encompassing four polynomials. By combining Equation (17) and Equation (18), the Bernstein polynomial can be derived as shown in Equation (19).

(19) $$\left\{\begin{array}{l} B_{0,3}(p)={\left(1-p\right)}^{3}\\ B_{1,3}(p)=3\cdot p\cdot {\left(1-p\right)}^{2}\\ B_{2,3}(p)=3\cdot p^{2}\cdot (1-p)\\ B_{3,3}(p)=p^{3} \end{array}\right.$$

**Figure 6:**
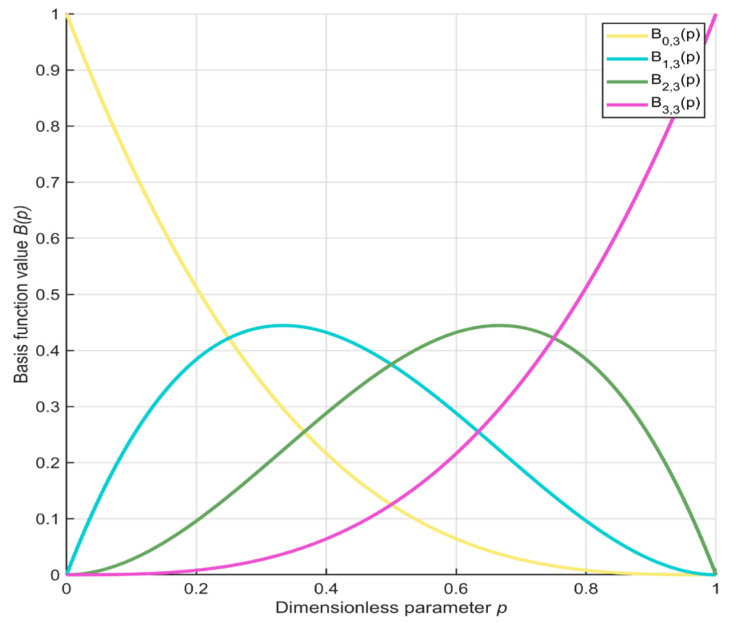
Third-order Bernstein polynomial.

To more intuitively illustrate the properties of the third-order Bernstein polynomial, Figure 6 displays its functional graph. As illustrated in the figure, when p varies within the interval [0, 1], the sum of the four polynomials remains consistently equal to 1. Leveraging this property, the optimal individual, suboptimal individual, and two randomly selected individuals from the population are weighted to generate weighted individuals. This process is expressed using Equation (20).

(20) $$X_{weight}=B_{0,3}(p)\cdot X_{best}+B_{1,3}(p)\cdot X_{be}+B_{2,3}(p)\cdot X_{rand1}+B_{3,3}(p)\cdot X_{rand2}$$

where, $X_{weight}$ denotes the weighted individual. $X_{be}$ is defined as a randomly selected individual from the subset comprising the top 20% highest-quality individuals in the population. $X_{rand1}$ and $X_{rand2}$ represent two distinct randomly selected individuals from the population. Subsequently, the generated weighted individual is used to guide other individuals, and this process is expressed using Equation (21).

(21) $$X_{i}^{t+1}=X_{i}^{t}+acrtan\left(2\cdot \pi \cdot \frac{t}{Max_{iter}}\right)\cdot (X_{weight}-X_{i}^{t})$$

**Figure 7:**
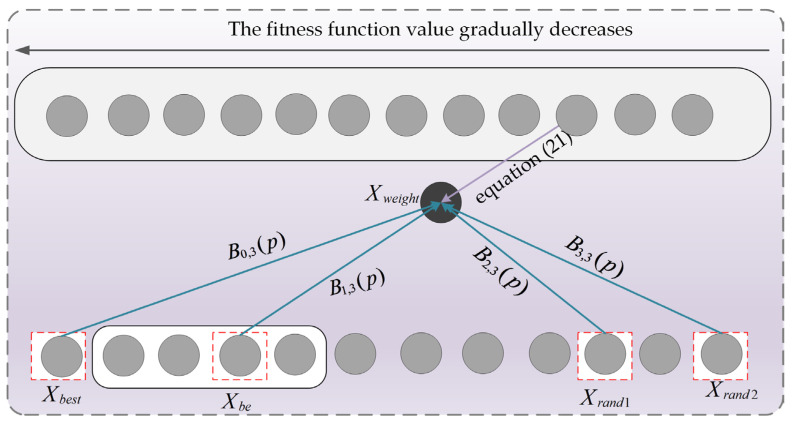
Third-order Bernstein guided strategy schematic.

To facilitate an intuitive understanding of the third-order Bernstein-guided strategy, Figure 7 presents a schematic diagram of its principles. By applying the third-order Bernstein-guided strategy to the population, the weighted properties of the Bernstein polynomial are effectively utilized, thereby enhancing the algorithm’s local exploitation capability and subsequently improving the quality of mural image segmentation.

### 3.4. Implementation of ANBPO

**Figure 8:**
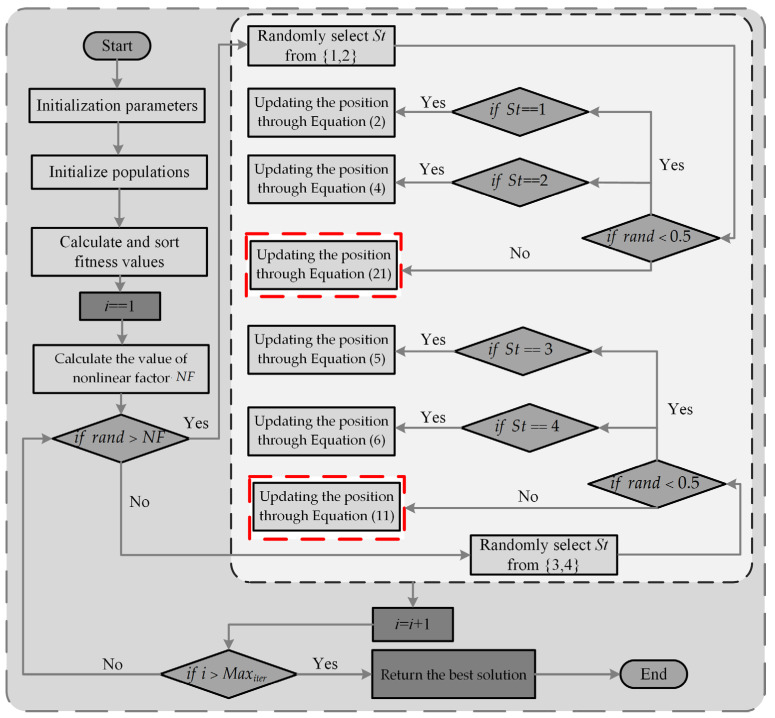
ANBPO process diagram.

The original PO exhibits deficiencies in global exploration capability, local exploitation capability, and an imbalance between the global exploration and local exploitation phases when solving mural image segmentation problems, resulting in suboptimal segmentation performance. To address these issues, this paper proposes an enhanced PO variant, termed ANBPO, by integrating an adaptive learning strategy, a nonlinear factor, and a third-order Bernstein-guided strategy. ANBPO effectively improves the algorithm’s capability for mural image segmentation, ensuring that the image is segmented effectively while preserving its structural and feature integrity. To facilitate an intuitive understanding of ANBPO’s execution logic, Algorithm 2 provides the pseudocode for its implementation, and Figure 8 illustrates the algorithm’s execution flowchart. In Figure 8, the improved strategies introduced in this study are highlighted with red dashed boxes. When solving the mural image segmentation problem, the process begins with population initialization. Subsequently, global exploration and local exploitation operations, marked by the indicator “St,” are performed to locate the optimal segmentation thresholds. Afterward, once the algorithm reaches the predefined maximum number of iterations, the optimal mural image segmentation thresholds are output, and the loop terminates.
**Algorithm 2:** Pseudo-code of ANBPO1: Initialization: N, ub, lb, dim$,\;Max_{iter}$2: Generate initialized population using Equation (1)3: ***for*** $i=1:Max_{iter}$ ***do***4:   Calculate fitness function value5:   Save the best individual6:   ***for*** j=1:N ***do***7:    Calculate the value of nonlinear factor NF8:    ***if*** rand>NF9:     ***if*** rand<0.510:      St=randi([1,2])11:      Foraging behavior12:      ***if*** St==1 ***Then***13:       Updating the individual using Equation (2)14:      Staying behavior15:      ***else if*** St==2 ***Then***16:       Updating the individual using Equation (4)17:      ***end if***18:     ***else***19:      Updating the individual using Equation (21)20:     ***end if***21:    ***Else***22:     ***if*** rand<0.523:      St=randi([3,4])24:      Communicating behavior25:      ***if*** St==3 ***Then***26:       Updating the individual using Equation (5)27:      Fear of strangers’ behavior28:      ***else if*** St==429:       Updating the individual using Equation (6)30:      ***end*** ***if***31:     ***else***32:      Updating the individual using Equation (11)33:     ***end if***34:    ***end if***35:   ***end*** ***for***36:  ***end for***37: Return the best individual


## 4. Experimental Results on Mural Image Segmentation

**Figure 9:**
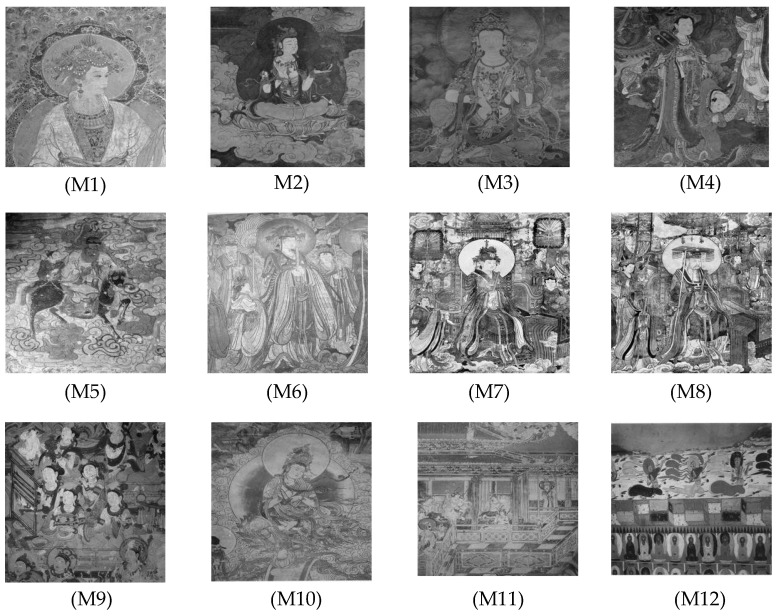
Mural image information.

This section primarily evaluates the performance of the proposed ANBPO for mural image segmentation. Specifically, as illustrated in Figure 9, experiments were conducted on twelve mural images sourced from an open-source standard mural image segmentation dataset. This dataset is accessible via the link: https://ww2.mathworks.cn/matlabcentral/fileexchange/181489-mural-image-segmentation-dataset (accessed on 19 July 2025). It primarily comprises notable examples of mural images and has undergone image standardization processing, making it effectively suitable for performance testing in mural image segmentation tasks. To comprehensively assess the algorithm’s performance, ANBPO was compared with 5 state-of-the-art algorithms, and the parameter settings for these 5 comparative algorithms are detailed in Table 1. In each experiment, the algorithm was independently run 30 times, with a population size of 40 and a maximum number of iterations set to 100. A comprehensive evaluation of ANBPO’s mural image segmentation performance was conducted by analyzing population diversity, the exploration/exploitation ratio, fitness function values, convergence behavior, peak signal-to-noise ratio (PSNR) [36], structural similarity (SSIM) [37], and feature similarity (FSIM) [38]. To ensure experimental fairness, all experimental codes involved in this study were developed and executed using MATLAB 2022 Rb on the Windows 11 operating system. The hardware configuration included 32 GB of RAM and an Intel(R) Core (TM) Ultra 5 processor.

The following section will provide a detailed introduction to the calculation methods for the PSNR, SSIM, and FSIM metrics. First, the PSNR metric is computed using Equation (22).

(22) $$PSNR=10\log_{10}\left(\frac{255^{2}}{MSE}\right)$$

where, MSE represents the mean squared error between the original image I and the segmented image $I^{\prime }$, and it is calculated using Equation (23).

(23) $$MSE=\frac{1}{MN}\sum_{j=1}^{M}\sum_{k=1}^{N}{\left[I(j,k)-I^{\prime }(j,k)\right]}^{2}$$

where, MN denotes the size of the image, I(j,k) is the grayscale value of the original image at position (j,k), while $I^{\prime }(j,k)$ is the grayscale value of the segmented image at the same position (j,k). SSIM is a method for measuring the similarity between two images and is computed using Equation (24).

(24) $$\mathrm{SSIM}\left(I,I^{\prime }\right)=\frac{\left(2\mu_{I}\mu_{I^{\prime }}+C_{1})(2\sigma_{II^{\prime }}+C_{2}\right)}{\left(\mu_{I}^{2}+\mu_{I^{\prime }}^{2}+C_{1})(\sigma_{I}^{2}+\sigma_{I^{\prime }}^{2}+C_{2}\right)}$$

where, $\mu_{I}$ and $\mu_{I^{\prime }}$ represent the mean grayscale values of the original image and the segmented image, respectively. $\sigma_{II^{\prime }}$ denotes the covariance between the grayscale values of the original image and the segmented image. $\sigma_{I}^{2}$ is the variance of the grayscale values in the original image, while $\sigma_{I^{\prime }}^{2}$ is the variance of the grayscale values in the segmented image. The constants are defined as $C_{1}={\left(k_{1}L\right)}^{2}$ and $C_{1}={\left(k_{2}L\right)}^{2}$, with typical values of L=256, $k_{1}=0.01$, and $k_{2}=0.03$. Finally, FSIM is calculated using Equation (25).

(25) $$\mathrm{FSIM}\left(I,I^{\prime }\right)=\frac{\sum_{x\in \Omega }S_{L}(x)\cdot PC_{m}(x)}{\sum_{x\in \Omega }PC_{m}(x)}$$

where, x represents a pixel, and Ω denotes the entire spatial domain of the image. $PC_{m}=\max(PC_{1},PC_{2})$, where $PC_{1}$ and $PC_{2}$ are the phase congruency values of the original image and the segmented image, respectively.

### 4.1. The Concept of Otsu Segmentation Technique

This section primarily focuses on modeling the fitness function for the mural image segmentation problem. In this study, the Otsu image segmentation technique is employed to segment mural images. The core idea of Otsu’s method is to achieve image segmentation by maximizing the inter-class variance between different regions of the image. Therefore, when using ANBPO to search for optimal segmentation threshold combinations, the inter-class variance between different image regions is adopted as the objective function. Below, the concept of the Otsu image segmentation technique is introduced in detail. First, assume that the grayscale pixel matrix of the image to be segmented is denoted as I, and the image contains L grayscale levels. Let $n_{i}$ represent the number of pixels with grayscale level i in the image. Based on these assumptions, the total number of pixels N in the image I is calculated using Equation (26).

(26) $$N=\sum_{i=0}^{L-1}n_{i}$$

Subsequently, the pixel proportion $P_{i}$ of grayscale level i in the entire image I is calculated using Equation (27).

(27) $$P_{i}=\frac{n_{i}}{N},\;\;i=0,1,\ldots ,L-1$$

where, $P_{i}\geq 0$, and $P_{0}+P_{1}+\ldots +P_{L-1}=1$. Let the number of thresholds for image segmentation be k, and assume the segmentation threshold is t. The image can then be divided into two regions based on threshold t: The region with pixel grayscale values in the interval [1,t] is classified as the object region, and the region with pixel grayscale values in the interval [t,L−1] is classified as the background region. Let the ratio of the number of pixels in the object region to the total number of pixels in the image be $\omega_{0}$, the average pixel value of the object region be $\mu_{0}$, the ratio of the number of pixels in the background region to the total number of pixels be $\omega_{1}$, and the average pixel value of the background region be $\mu_{1}$. The average pixel value of the entire image is denoted as μ, and the variance between the different segmented regions is denoted as ν. Based on these assumptions, $\omega_{0}$, $\mu_{0}$, $\omega_{1}$, $\mu_{1}$, μ, and ν are calculated using Equations (28), (29), (30), (31), (32) and (33), respectively.

(28) $$\omega_{0}=\sum_{i=0}^{t}P_{i}$$

(29) $$\mu_{0}=\sum_{i=0}^{t}\frac{iP_{i}}{\omega_{0}}$$

(30) $$\omega_{1}=\sum_{i=t+1}^{L-1}P_{i}$$

(31) $$\mu_{1}=\sum_{i=t+1}^{L-1}\frac{iP_{i}}{\omega_{1}}$$

(32) $$\mu =\sum_{i=0}^{k-1}\omega_{i}\mu_{i}\;\;=\sum_{i=0}^{L-1}iP_{i}$$

(33) $$v(t)=\omega_{0}{\left(\mu_{0}-\mu \right)}^{2}+\omega_{1}{\left(\mu_{1}-\mu \right)}^{2}=\omega_{0}\omega_{1}{\left(\mu_{0}-\mu_{1}\right)}^{2}$$

Subsequently, the optimal segmentation threshold $t_{best}$ for the image is calculated using Equation (34).

(34) $$t_{\mathrm{best}}=\arg\max_{0\leq t\leq L}\left[v(t)\right]$$

Subsequently, the inter-class variance between different regions when the number of thresholds is k calculated using Equation (35).

(35) $$\begin{array}{c} v(t_{1},t_{2},\ldots ,t_{k})=\omega_{0}\omega_{1}{\left(\mu_{0}-\mu_{1}\right)}^{2}+\omega_{0}\omega_{2}{\left(\mu_{0}-\mu_{2}\right)}^{2}\;+\cdots \\ \;\;\;\;\;+\omega_{0}\omega_{k}{\left(\mu_{0}-\mu_{k}\right)}^{2}+\omega_{1}\omega_{2}{\left(\mu_{1}-\mu_{2}\right)}^{2}+\cdots \\ \;\;\;\;\;\;\;+\omega_{1}\omega_{3}{\left(\mu_{1}-\mu_{3}\right)}^{2}+\ldots +\omega_{k-1}\omega_{k}{\left(\mu_{k-1}-\mu_{k}\right)}^{2} \end{array}$$

where, $\omega_{i}$ and $\mu_{i}$ are calculated using Equation (36) and Equation (37), respectively.

(36) $$\omega_{i-1}=\sum_{i=t_{i-1}+1}^{t_{i}}P_{i},\;\;1\leq i\leq k+1$$

(37) $$\mu_{i-1}=\sum_{i=t_{i-1}+1}^{t_{i}}\frac{iP_{i}}{\omega_{i-1}},\;\;1\leq i\leq k+1$$

Assume the optimal segmentation threshold combination for the image is denoted as $T_{\mathrm{best}}=(t_{1}^{*},t_{2}^{*},\ldots ,t_{k}^{*})$. The optimal threshold combination $T_{\mathrm{best}}$ is then calculated using Equation (38).

(38) $$T_{\mathrm{best}}=\arg\max_{0\leq t_{1}\leq t_{2}\leq \ldots \leq \ldots \leq t_{k}}\left[v(t_{1},t_{2},\ldots ,t_{k})\right]$$

### 4.2. Discussion of Experimental Results

**Figure 10:**
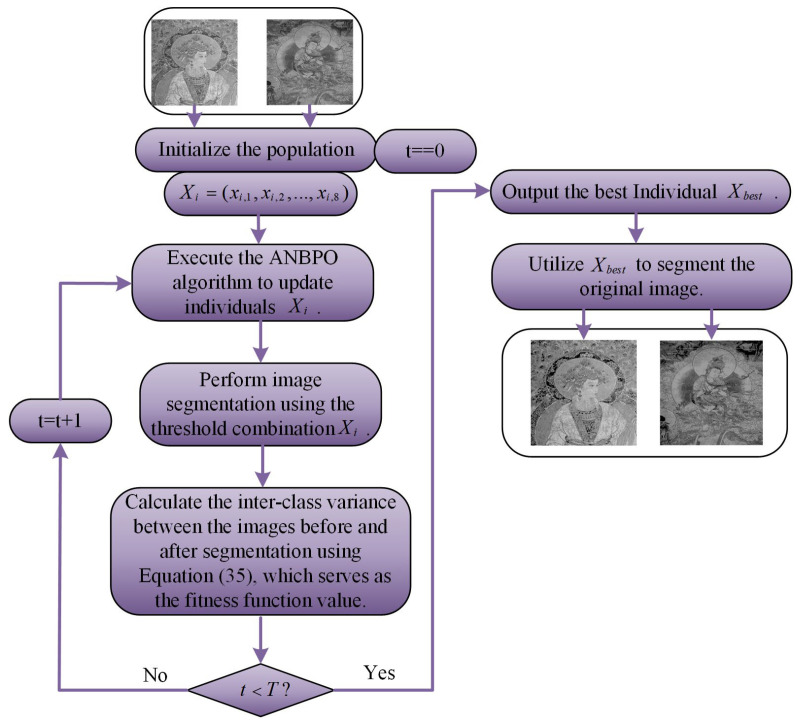
Using the ANBPO algorithm to solve mural image segmentation.

This section primarily focuses on analyzing the experimental results of applying ANBPO to mural image segmentation when the number of thresholds is set to 2, 4, 6, and 8. The specific image segmentation results are shown in Table 2. The analysis includes metrics such as population diversity, the exploration/exploitation ratio, fitness function values, convergence behavior, and image quality metrics: PSNR, SSIM, and FSIM. Before that, we need to sort out the logic of using the ANBPO algorithm to solve the problem of mural image segmentation. The simplified flowchart for solving the mural image segmentation problem using the ANBPO algorithm is shown in Figure 10. The specific steps are as follows:

**Step 1:** Based on the execution logic of the ANBPO algorithm, first initialize a population with N individuals. Among them, the ith individual is denoted as $X_{i}=(x_{i,1},x_{i,2},\ldots ,x_{i,8})$. Here, $X_{i}$ represents the ith threshold combination for mural image segmentation. Taking a case with eight segmentation thresholds as an example, the dimensionality of the individual $X_{i}$ is 8, indicating that the mural image needs to be segmented using eight thresholds. $x_{i,1}$ denotes the first threshold in the ith threshold combination.

**Step 2:** Segment the mural image according to the ith threshold combination $X_{i}$. Simultaneously, calculate the inter-class variance between the images before and after segmentation using Equation (35). This inter-class variance serves as the fitness function value in the ANBPO algorithm.

**Step 3:** Based on the fitness function values calculated for each individual in **Step 2**, determine the optimal threshold combination $X_{best}$ for mural image segmentation in the current iteration.

**Step 4:** Check whether the predefined maximum number of iterations has been reached. If the maximum number of iterations has been reached, terminate the algorithm, return $X_{best}$, and perform the final segmentation on the mural image to obtain the actual mural image segmentation results. Otherwise, proceed to **Step 5**.

**Step 5:** Update the population individuals according to the individual update process of the ANBPO algorithm, and then jump back to Step 2 for execution.

#### 4.2.1. Parameter Sensitivity Analysis

This section primarily conducts a sensitivity analysis of the operational parameters of ANBPO when solving mural image segmentation problems to determine the operational parameters for this experiment. Specifically, the operational parameters involved in the algorithm include the settings of population size and maximum number of iterations. To make reasonable selections for these two parameters, this section employs the control variable method for experimental investigations.

**Figure 11:**
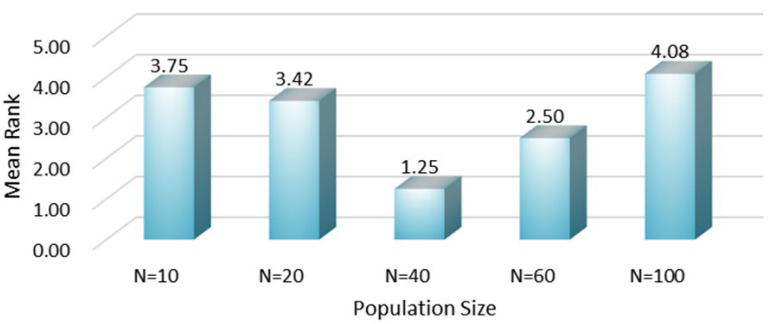
Average ranking under different population sizes.

Firstly, to determine the population size parameter, we defined five scenarios with population sizes of N=10, N=20, N=40, N=60, and N=100. With the maximum number of iterations fixed at 100, the average rankings of the fitness function values for 12 mural image segmentation problems are shown in Figure 11. As depicted in Figure 11, when the population size is set to 10 and 20, the algorithm’s average fitness values rank 3.75 and 3.42, respectively, indicating weaker performance compared to when the population size is 40. This is primarily because a smaller population search space makes the algorithm more prone to falling into local optima traps when solving mural image segmentation problems, unable to escape them, thereby affecting its image segmentation performance. Additionally, when the population size is set to 60 and 100, the algorithm’s average fitness values rank 2.50 and 4.08, respectively. In comparison, the algorithm performs best in mural image segmentation when the population size is set to 40. This is mainly because a larger population size enhances the algorithm’s search space, but its ability to exploit local regions is compromised, leading to a loss in precision during mural image segmentation. Therefore, setting the population size to 40 as an operational parameter for the ANBPO algorithm is deemed reasonable.

**Figure 12:**
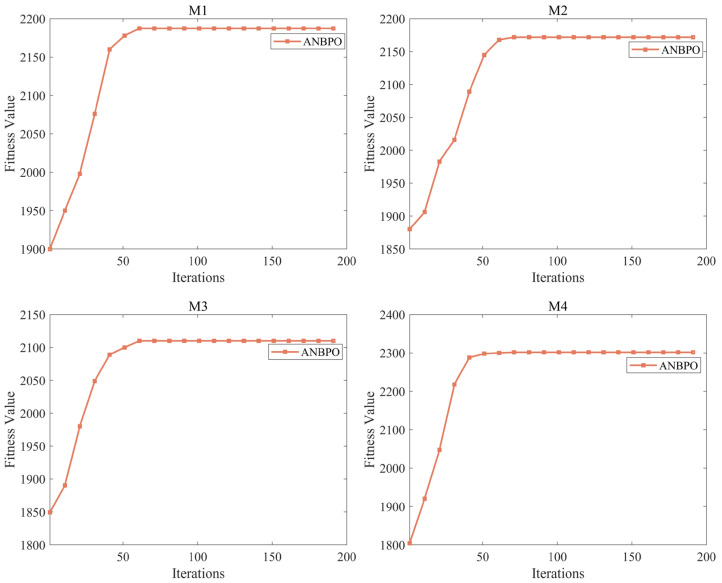
Convergence graph of the ANBPO algorithm with 200 iterations.

Subsequently, to determine an appropriate setting for the maximum number of iterations, we set the maximum number of iterations to 200 to observe the fitness function curve during mural image segmentation, with the number of segmentation thresholds set to 6. The fitness function variation curves for some mural image segmentation problems are shown in Figure 12. As seen in Figure 12, in most cases, the algorithm achieves a certain level of convergence after the 70th iteration. Subsequently, although there are still minor performance optimizations in subsequent iterations, the curve consistently follows a stable convergence trend. Therefore, to reduce computational costs during mural image segmentation, we set the maximum number of iterations to 100. Under this condition, the problem-solving process exhibits a stable trend, making it meaningful to analyze this phenomenon. In summary, we set the population size and maximum number of iterations to 40 and 100, respectively, as reasonable operational conditions for all subsequent experiments.

#### 4.2.2. Population Diversity Analysis

**Figure 13:**
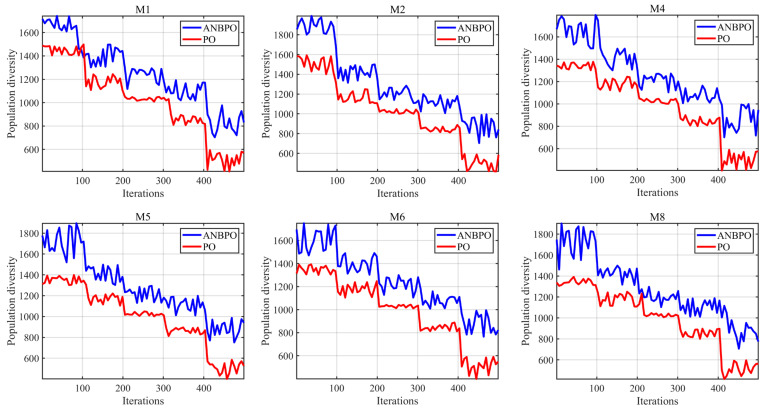
Population diversity.

This section analyzes the population diversity of ANBPO when applied to mural image segmentation. Experiments were conducted on 6 mural images, and the results are illustrated in Figure 13, where the number of iterations is set to 500, the number of segmentation thresholds is set to 6, the Y-axis represents real-time population diversity during algorithm execution, and the X-axis represents the iteration count. Generally, higher population diversity is beneficial for helping the algorithm escape local optima in threshold combinations and enhance global search capability, thereby improving the algorithm’s performance in mural image segmentation. As shown in the figure, under most conditions, the ANBPO algorithm maintains higher population diversity than the PO algorithm, facilitating broader exploration of the solution space and improving the quality of mural image segmentation. This is primarily attributed to the adaptive learning strategy and nonlinear factor proposed in this study, which effectively enhance the algorithm’s global exploration capability, leading to substantial improvements in population diversity.

#### 4.2.3. Exploration/Exploitation Ratio Analysis

**Figure 14:**
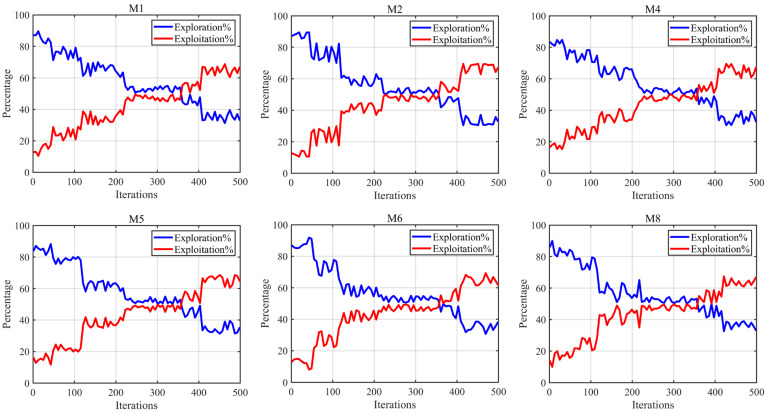
Exploration/exploitation ratio.

This section primarily analyzes the balance between the exploration and exploitation phases of ANBPO. Typically, a high-performance optimization algorithm first conducts global exploration of the solution space to locate potential optimal solution regions, followed by enhanced local exploitation to refine these regions and improve optimization precision. Thus, achieving a balance between exploration and exploitation is critical. The exploration/exploitation experimental results on 6 mural images are illustrated in Figure 14, where the number of segmentation thresholds is set to 6, the maximum number of iterations is set to 500, the blue line represents ANBPO’s global exploration rate, and the red line represents its local exploitation rate. As shown in the figure, during the early iterations, the global exploration phase dominates, enabling ANBPO to identify more potential optimal threshold combination regions. This is primarily attributed to the adaptive learning strategy proposed in this study, which effectively enhances the algorithm’s global exploration capability. Additionally, the algorithm retains sufficient local exploitation capability to improve optimization precision. As iterations progress, during the mid-stage iterations, the global exploration and local exploitation phases achieve balance, largely due to the nonlinear factor introduced in this study, which effectively balances the two phases and ensures a favorable trade-off across multiple metrics, thereby improving the algorithm’s ability to escape local optima in threshold combinations. During the late iterations, the local exploitation phase dominates, enabling the algorithm to enhance image segmentation precision. This is primarily due to the third-order Bernstein-guided strategy, which significantly strengthens the algorithm’s local exploitation capability. Furthermore, even in the late iterations, ANBPO maintains a certain level of global exploration capability due to the superiority of its strategies, aiding in escaping local optima in threshold combinations. In summary, the proposed ANBPO achieves a robust balance between the global exploration and local exploitation phases, effectively enhancing the quality of mural image segmentation.

#### 4.2.4. Effectiveness of Strategies Analysis

In this section, we primarily analyze the enhancing effects of the adaptive learning strategy, nonlinear factor, and third-order Bernstein-guided strategy proposed in this study on the performance of the PO algorithm, aiming to validate the effectiveness of these three learning strategies. Specifically, we define the APO algorithm by incorporating the adaptive learning strategy into the PO algorithm, the NPO algorithm by introducing the nonlinear factor into the PO algorithm, and the BPO algorithm by employing the third-order Bernstein-guided strategy within the PO algorithm. Subsequently, we apply the PO, APO, NPO, BPO, and ANBPO algorithms to solve 12 mural image segmentation problems and statistically rank their average fitness values to verify the effectiveness of the proposed strategies.

**Figure 15:**
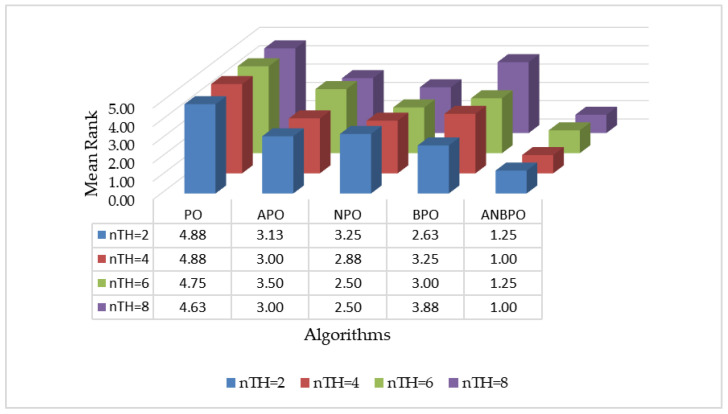
Average ranking of algorithms under different strategy guidance.

The experimental results are illustrated in Figure 15. As depicted in Figure 15, under four different numbers of segmentation thresholds, the average rankings of the APO, NPO, and BPO algorithms are all superior to those of the PO algorithm. This indicates that the three improvement strategies introduced in this study—the adaptive learning strategy, nonlinear factor, and third-order Bernstein-guided strategy—effectively enhance the mural image segmentation performance of the PO algorithm, confirming the validity of each strategy. Furthermore, the ANBPO algorithm, which incorporates all three learning strategies simultaneously, outperforms the APO, NPO, and BPO algorithms in terms of average ranking. This suggests that when these three improvement strategies are simultaneously introduced into the PO algorithm, they collectively enhance the algorithm’s global exploration capability, local exploitation capability, and overall balance, thereby further improving its performance. In summary, our study confirms that the three learning strategies introduced herein play a significant role in enhancing algorithm performance, and the simultaneous integration of these three strategies can further elevate the mural image segmentation performance of the algorithm.

#### 4.2.5. Fitness Function Values Analysis

**Figure 16:**
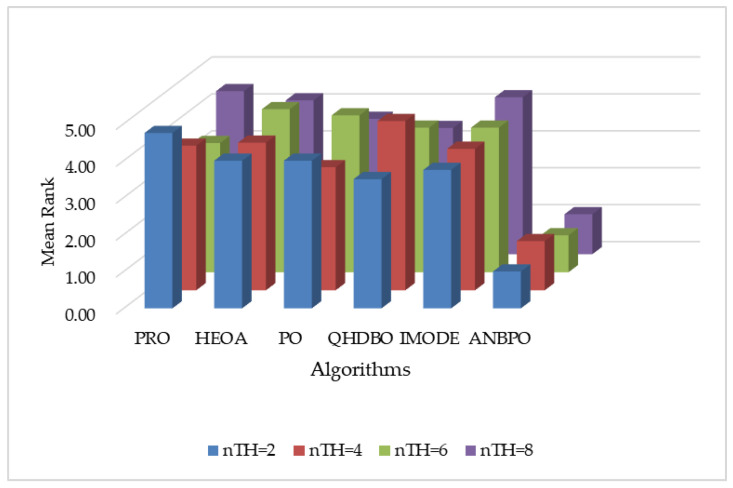
Ranking of fitness function values.

This section analyzes the fitness function values obtained by ANBPO for mural image segmentation with thresholds set to 2, 4, 6, and 8, as presented in Table 3. Specifically, “Mean” represents the average fitness function value from 30 independent experimental runs, “Std” denotes the standard deviation of these results, “Friedman” indicates the algorithm’s Friedman rank, and “Final Rank” reflects its overall performance ranking based on the Friedman metric. To visually demonstrate the advantages of ANBPO, Figure 16 illustrates the average fitness function value rankings of the algorithm across different numbers of segmentation thresholds.

As shown in the table, when the number of segmentation thresholds is 2 or 6, ANBPO achieves the optimal fitness function values across all 12 mural images, with a winning rate of 100%, demonstrating significant advantages in mural image segmentation. This performance is primarily attributed to the adaptive learning strategy and the third-order Bernstein-guided strategy proposed in this study, which effectively enhance the algorithm’s global exploration and exploitation capabilities, enabling comprehensive search and precise localization within the threshold combination space, and thereby improving the quality of mural image segmentation. Additionally, the incorporation of nonlinear factors balances the exploration and exploitation phases of the algorithm, enabling it to avoid local optima in threshold selection and enhancing segmentation accuracy. When the number of thresholds is 4 or 8, ANBPO attains the optimal fitness function values in 11 out of the 12 mural images, achieving a winning rate of 91.6%, indicating strong segmentation performance. However, it must be acknowledged that ANBPO’s performance occasionally lags behind that of the comparison algorithms, suggesting room for further improvement. Meanwhile, ANBPO achieves a Friedman ranking of 1.10, which is 71.1% higher than that of the second-ranked PO algorithm. Figure 16 visually confirms that, under varying numbers of segmentation thresholds, ANBPO consistently exhibits lower bar heights, validating its superior mural segmentation performance. Analysis of the fitness function values indicates that, owing to ANBPO’s advanced search strategies, it achieves higher mural segmentation performance compared to the comparison algorithms and can be considered a promising approach for mural image segmentation.

#### 4.2.6. Wilcoxon Rank Sum Test

The previous subsection analyzed the numerical results of the ANBPO algorithm for solving mural image segmentation problems. To comprehensively evaluate the performance of the ANBPO algorithm, this section conducts a Wilcoxon rank sum non-parametric test on the numerical experimental results of the fitness function, with a significance level set at 0.05. The experimental results are presented in Table 4, where “−“ indicates that the algorithm’s performance is significantly inferior to that of the ANBPO algorithm, “+” denotes that the algorithm’s performance is significantly superior to that of the ANBPO algorithm, and “=” signifies no significant difference in performance between the algorithm and the ANBPO algorithm.

As can be seen from the table, among the 48 experiments conducted, the PRO and QHDBO algorithms demonstrate significantly inferior performance compared to the ANBPO algorithm in 47 instances. The HEOA, PO, and IMODE algorithms show significantly inferior performance to the ANBPO algorithm in all 48 experiments. The ANBPO algorithm outperforms the comparative algorithms, with a winning ratio exceeding 95%. Therefore, it can be concluded that the ANBPO algorithm is a high-performing mural image segmentation algorithm. This advantage primarily stems from the introduction of the adaptive learning strategy, nonlinear factor, and third-order Bernstein-guided strategy in this study, which enhance the algorithm’s performance from the perspectives of global search, local exploitation, and algorithmic balance, thereby enabling the algorithm to achieve superior mural image segmentation performance.

#### 4.2.7. Convergence Analysis

**Figure 17:**
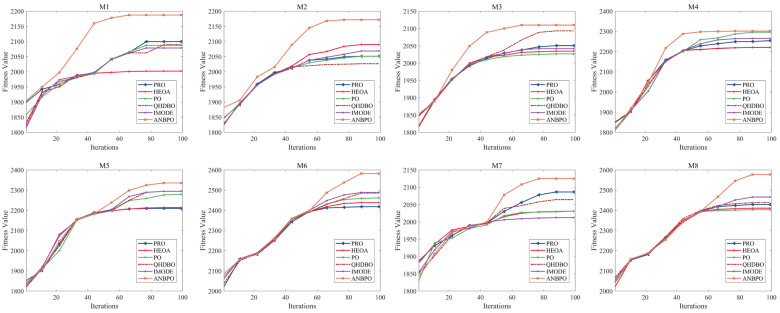
Convergence curve of fitness function value.

This section primarily analyzes the convergence behavior of ANBPO when solving the mural image segmentation problem. A high-quality algorithm should not only achieve superior optimization precision but also exhibit faster convergence speed. Figure 17 illustrates the convergence curves of the algorithms when the number of thresholds is set to 6, where the Y-axis represents the fitness function value and the X-axis represents the iteration count. As shown in the figure, all algorithms effectively segment the mural images, and their convergence curves stabilize. However, it is noteworthy that, compared to the competing algorithms, ANBPO gains a significant lead after the 40th iteration, and this advantage gradually widens until convergence. This demonstrates that ANBPO not only achieves higher convergence precision but also exhibits faster convergence speed compared to the competing algorithms. Consequently, ANBPO can be regarded as a highly applicable method for mural image segmentation.

#### 4.2.8. PSNR Analysis

**Figure 18:**
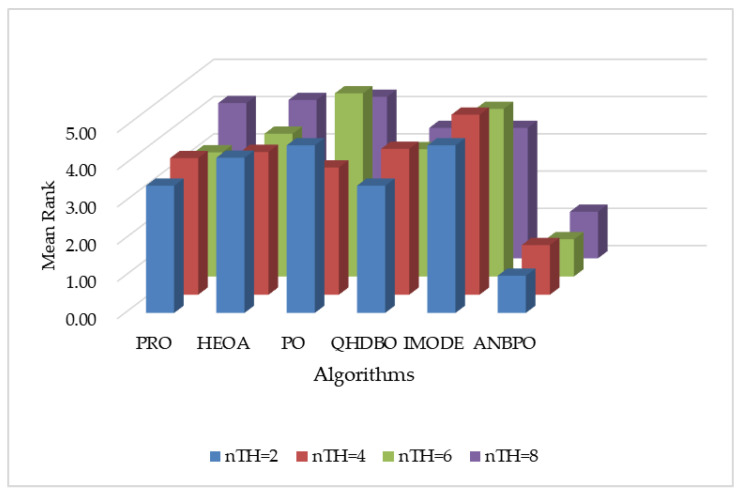
Ranking of PSNR values.

This section primarily analyzes the PSNR performance of ANBPO for mural image segmentation. Table 5 presents the PSNR values obtained for segmentation thresholds of 2, 4, 6, and 8. Here, “Mean” denotes the average PSNR value across 30 independent and non-repetitive experimental runs, “Std” represents the standard deviation of these results, “Friedman” indicates the algorithm’s Friedman rank, and “Final Rank” reflects the algorithm’s ultimate ranking based on the Friedman metric. To visually demonstrate ANBPO’s superiority in terms of PSNR, Figure 18 illustrates the average PSNR rankings of the algorithm across different numbers of segmentation thresholds.

As shown in the table, when the number of segmentation thresholds is set to 2 or 6, ANBPO achieves the optimal Peak Signal-to-Noise Ratio (PSNR) values across all 12 mural images, with a winning rate of 100%, indicating minimal distortion in mural image segmentation. This performance is primarily attributed to the adaptive learning strategy proposed in this study, which effectively enhances the algorithm’s global exploration capability, minimizing noise interference during mural segmentation and preserving the quality of the segmented images. Additionally, the third-order Bernstein-guided strategy strengthens the algorithm’s local exploitation ability, significantly improving the quality of threshold combinations and enhancing segmentation quality. Furthermore, the integration of nonlinear factors achieves a better balance between the global search and local exploitation phases of the algorithm, bolstering its image denoising capability and improving segmentation accuracy. When the number of thresholds is 4 or 8, ANBPO attains the optimal PSNR values in 11 out of the 12 mural images, achieving a winning rate of 91.6%, demonstrating robust segmentation performance. Although ANBPO occasionally underperforms compared to competing algorithms, its overall PSNR performance is superior. ANBPO achieves a Friedman ranking of 1.15, which is 67.6% higher than that of the second-ranked QHDBO algorithm. Figure 18 visually confirms that, under varying numbers of segmentation thresholds, ANBPO exhibits a relatively low average PSNR ranking, validating its low distortion rate in segmented images. Analysis of the PSNR values indicates that, owing to ANBPO’s enhanced exploration and exploitation capabilities, it achieves minimal image distortion in mural segmentation tasks, ensuring high-quality segmentation. Therefore, ANBPO can be considered a promising approach for mural image segmentation.

#### 4.2.9. SSIM Analysis

**Figure 19:**
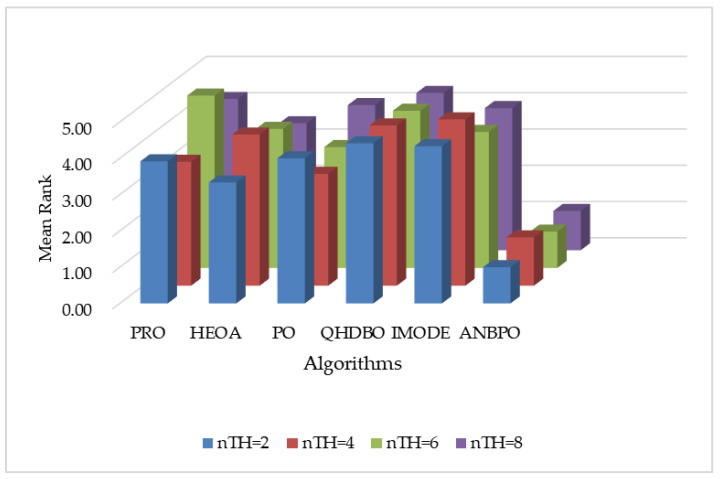
Ranking of SSIM values.

This section primarily analyzes the SSIM performance of ANBPO for mural image segmentation. Table 6 presents the SSIM values obtained for segmentation thresholds of 2, 4, 6, and 8. Here, “Mean” denotes the average SSIM value across 30 independent and non-repetitive experimental runs, “Std” represents the standard deviation of these results, “Friedman” indicates the algorithm’s Friedman rank, and “Final Rank” reflects the algorithm’s ultimate ranking based on the Friedman metric. To visually demonstrate ANBPO’s superiority in terms of SSIM, Figure 19 illustrates the average SSIM rankings of the algorithm across different numbers of segmentation thresholds.

As shown in the table, when the number of segmentation thresholds is set to 2 or 6, ANBPO achieves the optimal Structural Similarity Index (SSIM) values across all 12 mural images, with a winning rate of 100%. This indicates a high degree of structural similarity in the segmented images and demonstrates the preservation of most structural features from the original images. This performance is primarily attributable to the adaptive learning strategy proposed in this study, which effectively enhances the algorithm’s global exploration capability, maximizes the preservation of structural integrity during mural image segmentation, and retains original image information throughout the effective segmentation process. Additionally, the third-order Bernstein-guided strategy strengthens the algorithm’s local exploitation ability, significantly improving the quality of threshold combinations for image segmentation and ensuring the retention of structural information. Furthermore, the integration of nonlinear factors achieves a better balance between the global search and local exploitation phases of the algorithm, enhancing its multi-objective optimization capability and generating a comprehensive optimal segmentation solution that preserves most of the structural information from the original images. When the number of thresholds is 4 or 8, ANBPO attains the optimal SSIM values in 11 out of the 12 mural images, achieving a winning rate of 91.6%, demonstrating robust structural preservation capability. Although ANBPO occasionally underperforms in terms of SSIM compared to competing algorithms, its overall SSIM performance is superior. ANBPO achieves a Friedman ranking of 1.10, which is 69.4% higher than that of the second-ranked PO algorithm. Figure 19 visually confirms that, under varying numbers of segmentation thresholds, ANBPO exhibits a relatively low average SSIM ranking, validating its higher feature preservation capability in segmented images. Analysis of the SSIM values indicates that, owing to ANBPO’s enhanced ability to balance across multiple metrics, it achieves exceptional feature preservation performance in mural image segmentation tasks, ensuring high-quality segmentation. Therefore, ANBPO can be considered a promising approach for mural image segmentation.

#### 4.2.10. FSIM Analysis

**Figure 20:**
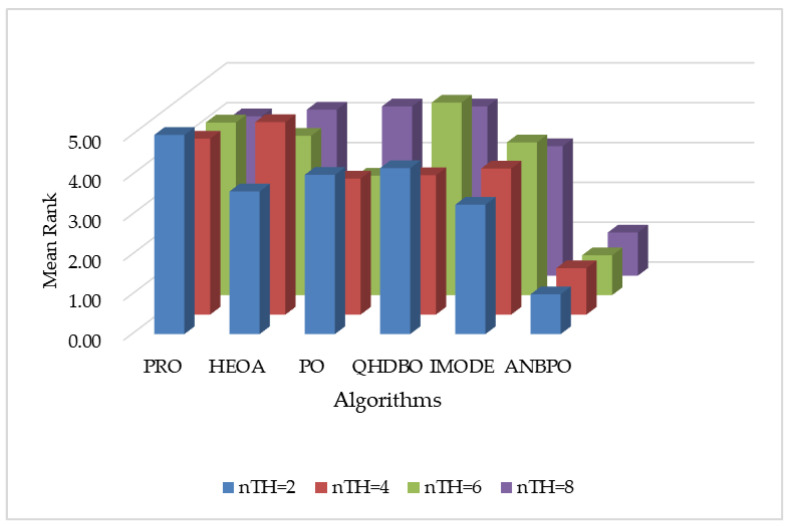
Ranking of FSIM values.

This section primarily analyzes the FSIM performance of ANBPO for mural image segmentation. Table 7 presents the FSIM values obtained for segmentation thresholds of 2, 4, 6, and 8. Here, “Mean” denotes the average FSIM value across 30 independent and non-repetitive experimental runs, “Std” represents the standard deviation of these results, “Friedman” indicates the algorithm’s Friedman rank, and “Final Rank” reflects the algorithm’s ultimate ranking based on the Friedman metric. To visually demonstrate ANBPO’s superiority in terms of FSIM, Figure 20 illustrates the average FSIM rankings of the algorithm across different numbers of segmentation thresholds.

As shown in the table, when the number of segmentation thresholds is set to 2 or 6, ANBPO achieves the optimal Feature Similarity Index (FSIM) values across all 12 mural images, with a winning rate of 100%. This demonstrates high feature similarity and enhanced visual similarity in the segmented images, thereby reducing distortion levels. This performance is primarily attributable to the adaptive learning strategy proposed in this study, which effectively enhances the algorithm’s global exploration capability, enabling better capture of visual features and minimizing distortion during image segmentation. Additionally, the third-order Bernstein-guided strategy strengthens the algorithm’s local exploitation ability, significantly improving the quality of threshold combinations for mural image segmentation and effectively preserving visual feature information from the original images, thereby enhancing segmentation quality. Furthermore, the integration of nonlinear factors achieves a better balance between the global search and local exploitation phases of the algorithm, effectively balancing multiple metrics in image segmentation to ensure the preservation of structural and visual features. When the number of thresholds is 4 or 8, ANBPO attains the optimal FSIM values in 11 out of the 12 mural images, achieving a winning rate of 91.6%. This demonstrates strong visual feature preservation capability and reduces distortion in the segmented images. Although ANBPO occasionally underperforms in terms of FSIM compared to competing algorithms, its overall FSIM performance is superior. ANBPO achieves a Friedman ranking of 1.06, which is 69.7% higher than that of the second-ranked IMODE algorithm. Figure 20 visually confirms that, under varying numbers of segmentation thresholds, ANBPO exhibits a relatively low average FSIM ranking, proving its higher visual feature capture capability in segmented images. Analysis of the FSIM values indicates that, compared to competing algorithms, ANBPO effectively segments mural images while maximally preserving original visual features, improving segmentation quality, and reducing distortion, making it a promising approach for mural image segmentation.

#### 4.2.11. Runtime Analysis

This section primarily focuses on analyzing the runtime of the ANBPO algorithm when solving mural image segmentation problems. The experimental results are presented in Table 8, where “Mean Rank” indicates the average runtime ranking of the algorithm across 12 mural image segmentation problems, and “Final Rank” represents the algorithm’s final ranking based on the “Mean Rank” metric.

**Figure 21:**
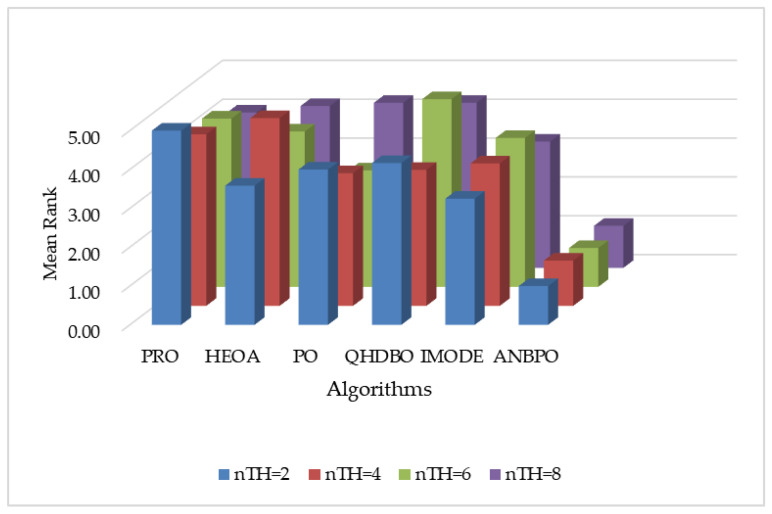
Average ranking of runtime algorithm.

As shown in Table 8, in 43 out of the 48 experiments conducted, the ANBPO algorithm achieved shorter runtimes compared to the competing algorithms, with a winning rate of 89.5%. This is primarily attributed to the ANBPO algorithm’s simpler operational structure, which reduces the time consumed during actual computations. Additionally, as indicated in the last row of the table, the ANBPO algorithm attained an average runtime ranking of 1.13, demonstrating its superiority over the competing algorithms. To visually compare the algorithms’ runtimes, Figure 21 displays a bar chart of the average runtime rankings under different numbers of segmentation thresholds. From the figure, it is evident that the ANBPO algorithm consistently exhibits lower runtime costs under all four operational conditions. In summary, due to its concise operational logic, the ANBPO algorithm holds an advantage in terms of runtime metrics, making it a practical and effective mural image segmentation algorithm.

#### 4.2.12. Comprehensive Analysis

**Figure 22:**
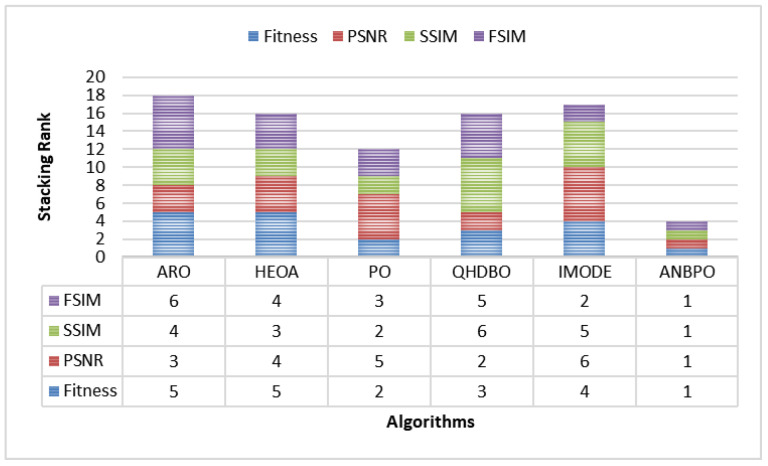
Stacking rank.

The preceding sections conducted separate analyses of the fitness function value, PSNR, SSIM, and FSIM metrics. This section aims to provide a comprehensive analysis of these four indicators. The experimental results are shown in Figure 22, where the *Y*-axis represents the stacked ranking of the fitness function value, PSNR, SSIM, and FSIM. As depicted, the ANBPO algorithm achieves a final ranking of 1 across all four metrics fitness function value, PSNR, SSIM, and FSIM attaining the lowest stacked bar height of 4. Compared to competing algorithms, it demonstrates superior overall performance in mural image segmentation. This is primarily attributed to the three learning strategies proposed in this study, which enable the algorithm to effectively balance multiple metrics and determine a globally optimal threshold segmentation scheme. Consequently, ANBPO ensures high-quality segmentation of mural images, validating its effectiveness as an efficient image segmentation method.

## 5. Conclusions and Future Works

Due to the insufficient data support in existing neural network-based image segmentation methods, the trained image segmentation models exhibit certain limitations, thereby adversely affecting the performance of mural image segmentation. Consequently, optimization algorithm-based image segmentation methods have gradually gained popularity. This study addresses the issue of PO easily falling into local optimal segmentation threshold traps when solving mural image segmentation problems, thereby degrading the quality of the segmented images. The root cause lies in PO’s deficiencies, including insufficient exploration capability, inadequate exploitation capability, and imbalance between the exploration and exploitation phases. To mitigate these limitations and improve mural image segmentation quality, this section proposes an enhanced PO variant, termed ANBPO, by integrating an adaptive learning strategy, a nonlinear factor, and a third-order Bernstein-guided strategy into the PO framework. First, to address PO’s limited global exploration capability, an adaptive learning strategy is introduced to effectively enhance the algorithm’s global exploration, enabling it to thoroughly search the solution space and improve mural image segmentation quality. Second, to resolve the imbalance between global exploration and local exploitation, a nonlinear factor is proposed to balance these two phases, thereby strengthening the algorithm’s ability to escape local optimal segmentation thresholds and enhancing the segmentation quality of mural images. Finally, to counter PO’s inadequate exploitation capability, a third-order Bernstein-guided strategy is introduced to boost the algorithm’s local exploitation capability, improving the precision of mural image segmentation. Subsequently, the proposed ANBPO was applied to 8 mural image segmentation problems. Experimental results demonstrate that, compared to competing algorithms, ANBPO achieves a 93.7% win rate in terms of fitness function values, indicating superior segmentation performance. Additionally, ANBPO outperforms competing algorithms by 63.9%, 68.3%, and 69.12% on the PSNR, SSIM, and FSIM metrics, respectively. These results confirm that ANBPO maximally preserves the original feature information of mural images during segmentation, thereby enhancing the quality of mural image segmentation.

Despite ANBPO achieving exceptional overall performance in mural image segmentation, its performance may still exhibit limitations in certain specialized scenarios. Future work will focus on the following directions: (1). Developing more efficient search strategies based on ANBPO to further enhance the quality of mural image segmentation. (2). Extending ANBPO to solve more complex combinatorial optimization problems, thereby broadening its application domains. (3). Proposing a multi-objective version of ANBPO to address a wider range of complex optimization problems.

## Figures and Tables

**Table 1:** Baseline algorithms information.

Algorithms	Year	Parameter Settings
PRO [39]	2024	$\tau =\left(\frac{t}{T}\right)$
HEOA [40]	2024	$\omega =0.2\cdot \cos\left(\frac{\pi }{2}\left(1-\frac{t}{T}\right)\right)$
PO [33]	2024	No Parameters
QHDBO [41]	2024	RDB=6, EFDBO=13, SDB=11
IMODE [42]	2020	D=2, arch_rate=2.6

**Table 2:** Actual segmentation results of mural images.

Fun	nTH = 2	nTH = 4	nTH = 6	nTH = 8
M1	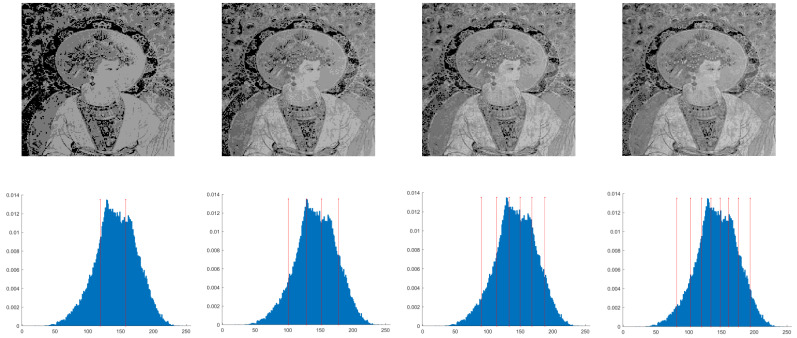			
M2	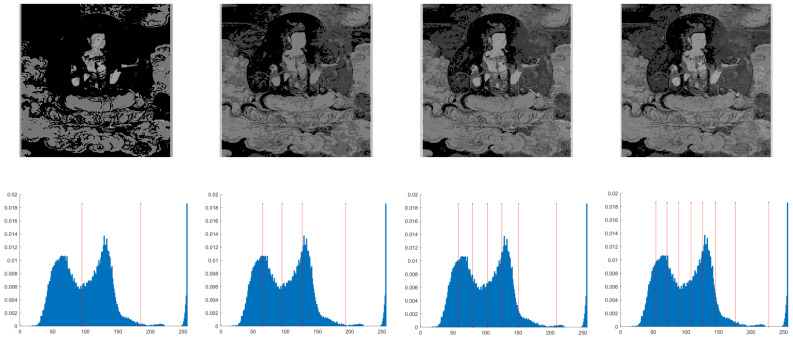			
M3	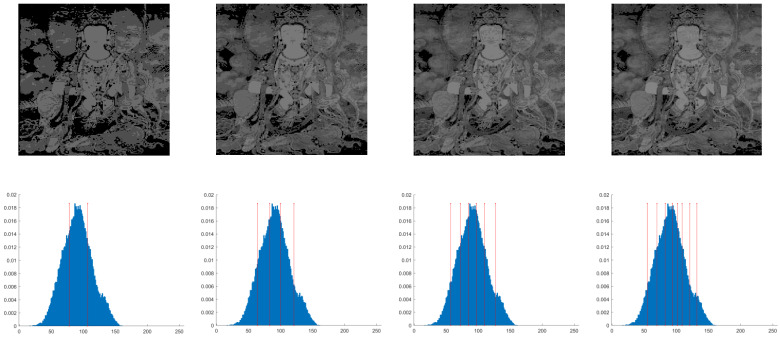			
M4	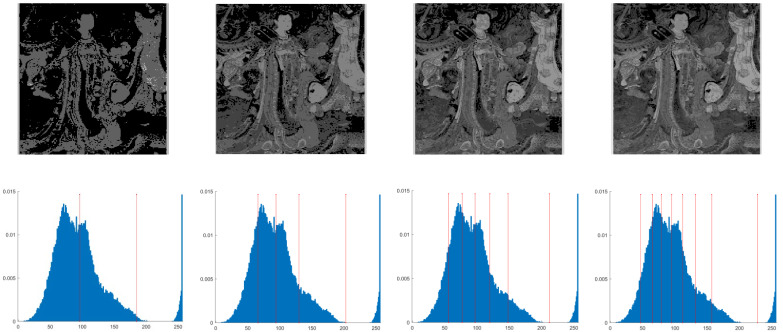			
M5	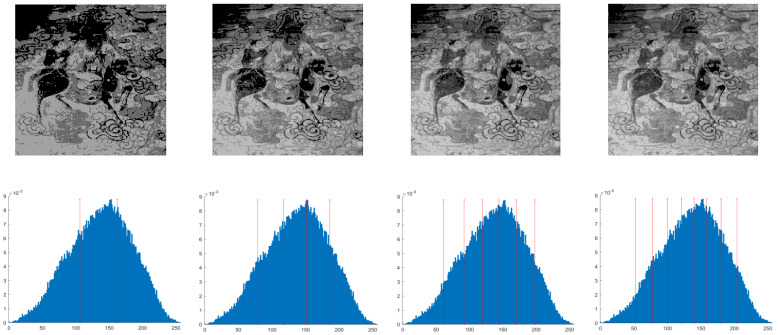			
M6	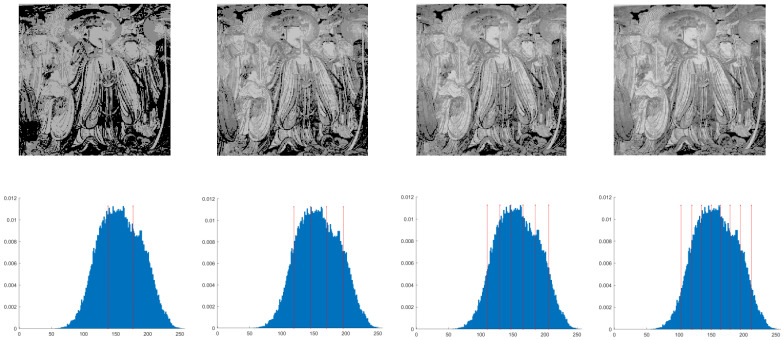			
M7	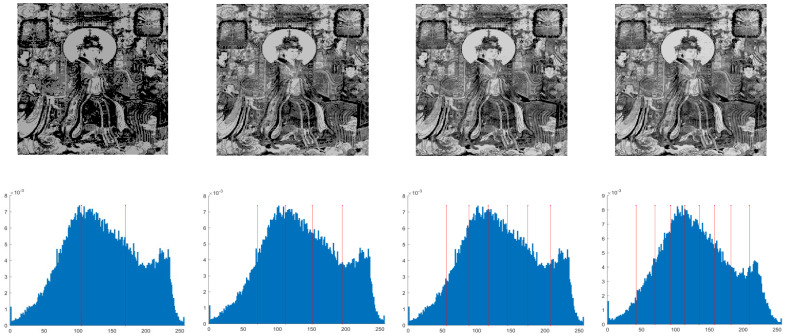			
M8	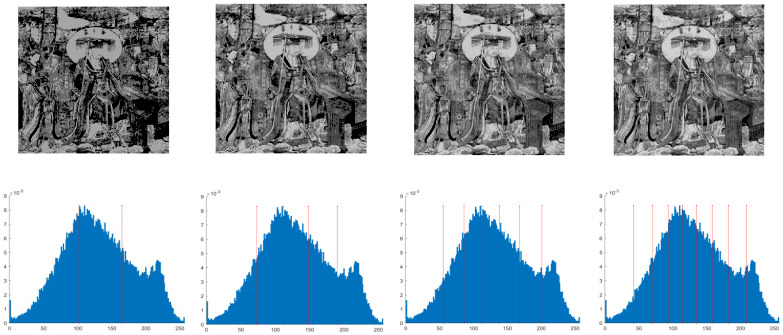			
M9	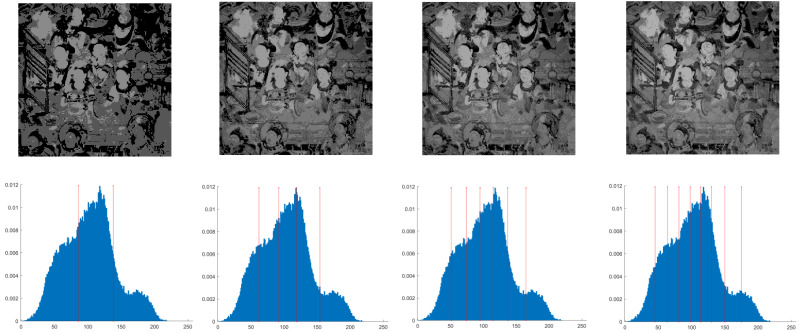			
M10	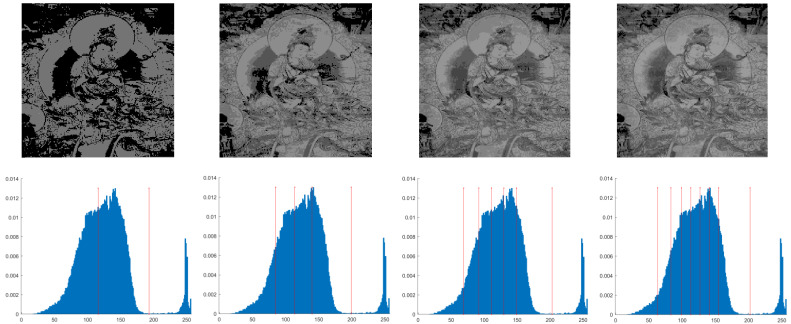			
M11	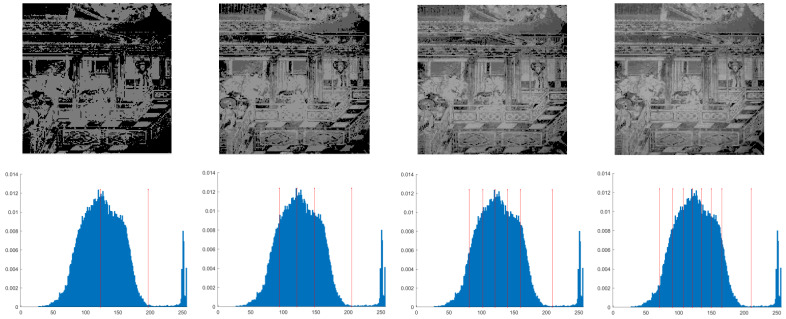			
M12	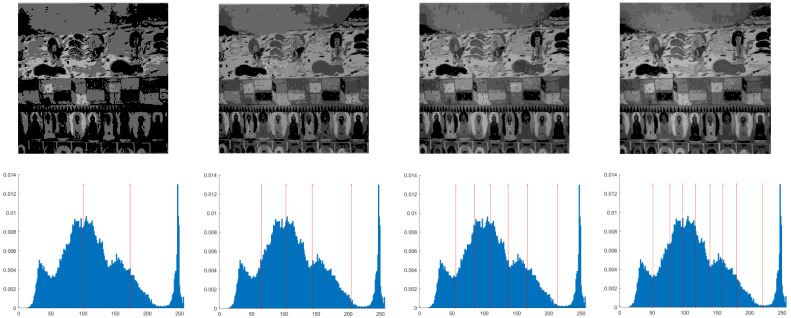			

**Table 3:** Fitness function value.

Fun	nTH	PRO		HEOA		PO		QHDBO		IMODE		ANBPO	
		Mean	Std	Mean	Std	Mean	Std	Mean	Std	Mean	Std	Mean	Std
M1	2	1.813 × 10^+03^	8.352 × 10^−01^	1.729 × 10^+03^	3.675 × 10^−01^	1.850 × 10^+03^	2.879 × 10^−01^	1.806 × 10^+03^	3.554 × 10^−01^	1.878 × 10^+03^	3.003 × 10^−01^	1.968 × 10^+03^	3.856 × 10^−03^
	4	1.960 × 10^+03^	3.968 × 10^−01^	1.974 × 10^+03^	4.176 × 10^−01^	1.977 × 10^+03^	5.018 × 10^−01^	1.914 × 10^+03^	2.319 × 10^−03^	1.957 × 10^+03^	9.386 × 10^−02^	2.055 × 10^+03^	6.688 × 10^−03^
	6	2.100 × 10^+03^	9.918 × 10^−01^	2.002 × 10^+03^	4.686 × 10^−01^	2.087 × 10^+03^	9.665 × 10^−01^	2.089 × 10^+03^	7.851 × 10^−01^	2.079 × 10^+03^	6.825 × 10^−01^	2.187 × 10^+03^	2.458 × 10^−03^
	8	2.137 × 10^+03^	1.707 × 10^−01^	2.125 × 10^+03^	6.466 × 10^−01^	2.171 × 10^+03^	3.681 × 10^−01^	2.175 × 10^+03^	6.164 × 10^−02^	2.177 × 10^+03^	9.645 × 10^−01^	2.281 × 10^+03^	4.850 × 10^−03^
M2	2	1.862 × 10^+03^	5.941 × 10^−01^	1.878 × 10^+03^	7.653 × 10^−01^	1.802 × 10^+03^	2.357 × 10^−01^	1.887 × 10^+03^	2.251 × 10^−01^	1.871 × 10^+03^	5.155 × 10^−01^	1.901 × 10^+03^	9.242 × 10^−03^
	4	1.998 × 10^+03^	1.871 × 10^−01^	1.958 × 10^+03^	5.132 × 10^−02^	1.994 × 10^+03^	7.300 × 10^−01^	1.927 × 10^+03^	1.509 × 10^−01^	1.959 × 10^+03^	8.456 × 10^−01^	2.036 × 10^+03^	8.011 × 10^−03^
	6	2.051 × 10^+03^	5.163 × 10^−01^	2.090 × 10^+03^	5.548 × 10^−02^	2.051 × 10^+03^	4.892 × 10^−01^	2.027 × 10^+03^	1.213 × 10^−01^	2.069 × 10^+03^	2.477 × 10^−01^	2.172 × 10^+03^	3.464 × 10^−03^
	8	2.161 × 10^+03^	5.903 × 10^−01^	2.188 × 10^+03^	3.963 × 10^−02^	2.177 × 10^+03^	2.244 × 10^−01^	2.187 × 10^+03^	7.401 × 10^−01^	2.161 × 10^+03^	1.428 × 10^−01^	2.281 × 10^+03^	1.847 × 10^−03^
M3	2	1.704 × 10^+03^	5.725 × 10^−01^	1.702 × 10^+03^	5.769 × 10^−01^	1.757 × 10^+03^	4.049 × 10^−01^	1.763 × 10^+03^	7.642 × 10^−01^	1.722 × 10^+03^	3.311 × 10^−01^	1.952 × 10^+03^	2.416 × 10^−03^
	4	1.910 × 10^+03^	4.311 × 10^−01^	1.961 × 10^+03^	5.306 × 10^−01^	1.943 × 10^+03^	2.542 × 10^−02^	1.945 × 10^+03^	3.695 × 10^−01^	1.967 × 10^+03^	2.824 × 10^−01^	2.069 × 10^+03^	2.703 × 10^−01^
	6	2.051 × 10^+03^	6.846 × 10^−01^	2.035 × 10^+03^	5.228 × 10^−01^	2.027 × 10^+03^	3.230 × 10^−01^	2.093 × 10^+03^	5.396 × 10^−01^	2.042 × 10^+03^	1.183 × 10^−01^	2.110 × 10^+03^	9.911 × 10^−01^
	8	2.107 × 10^+03^	7.664 × 10^−01^	2.111 × 10^+03^	5.327 × 10^−01^	2.107 × 10^+03^	5.773 × 10^−01^	2.160 × 10^+03^	5.689 × 10^−01^	2.104 × 10^+03^	7.490 × 10^−01^	2.230 × 10^+03^	9.125 × 10^−01^
M4	2	1.915 × 10^+03^	1.423 × 10^−01^	2.072 × 10^+03^	2.723 × 10^−01^	2.067 × 10^+03^	2.441 × 10^−01^	2.061 × 10^+03^	7.754 × 10^−02^	1.924 × 10^+03^	4.893 × 10^−01^	2.118 × 10^+03^	4.486 × 10^−03^
	4	2.109 × 10^+03^	5.008 × 10^−01^	2.198 × 10^+03^	6.679 × 10^−01^	2.138 × 10^+03^	2.440 × 10^−01^	2.106 × 10^+03^	1.184 × 10^−01^	2.161 × 10^+03^	6.653 × 10^−01^	2.212 × 10^+03^	3.122 × 10^−03^
	6	2.254 × 10^+03^	2.135 × 10^−01^	2.220 × 10^+03^	3.077 × 10^−01^	2.296 × 10^+03^	4.107 × 10^−01^	2.220 × 10^+03^	1.161 × 10^−01^	2.265 × 10^+03^	2.023 × 10^−02^	2.302 × 10^+03^	5.954 × 10^−03^
	8	2.340 × 10^+03^	6.163 × 10^−01^	2.359 × 10^+03^	6.875 × 10^−01^	2.316 × 10^+03^	7.406 × 10^−01^	2.357 × 10^+03^	3.099 × 10^−01^	2.326 × 10^+03^	9.467 × 10^−01^	2.488 × 10^+03^	7.470 × 10^−03^
M5	2	1.965 × 10^+03^	4.318 × 10^−01^	1.914 × 10^+03^	9.641 × 10^−01^	2.068 × 10^+03^	6.459 × 10^−01^	1.951 × 10^+03^	2.746 × 10^−02^	2.069 × 10^+03^	8.577 × 10^−01^	2.184 × 10^+03^	3.591 × 10^−03^
	4	2.183 × 10^+03^	9.105 × 10^−01^	2.168 × 10^+03^	4.511 × 10^−01^	2.122 × 10^+03^	9.968 × 10^−01^	2.154 × 10^+03^	1.662 × 10^−01^	2.141 × 10^+03^	6.976 × 10^−01^	2.140 × 10^+03^	3.207 × 10^−03^
	6	2.210 × 10^+03^	8.396 × 10^−01^	2.214 × 10^+03^	1.822 × 10^−01^	2.279 × 10^+03^	2.783 × 10^−02^	2.294 × 10^+03^	5.123 × 10^−01^	2.294 × 10^+03^	3.414 × 10^−01^	2.335 × 10^+03^	7.437 × 10^−03^
	8	2.380 × 10^+03^	3.985 × 10^−01^	2.379 × 10^+03^	4.268 × 10^−02^	2.397 × 10^+03^	8.447 × 10^−01^	2.385 × 10^+03^	8.494 × 10^−02^	2.355 × 10^+03^	8.251 × 10^−02^	2.446 × 10^+03^	5.010 × 10^−03^
M6	2	2.146 × 10^+03^	9.882 × 10^−01^	2.233 × 10^+03^	4.068 × 10^−01^	2.140 × 10^+03^	8.554 × 10^−01^	2.172 × 10^+03^	5.330 × 10^−01^	2.215 × 10^+03^	4.111 × 10^−01^	2.382 × 10^+03^	8.480 × 10^−03^
	4	2.321 × 10^+03^	3.821 × 10^−01^	2.327 × 10^+03^	1.676 × 10^−01^	2.385 × 10^+03^	7.740 × 10^−01^	2.363 × 10^+03^	5.560 × 10^−01^	2.390 × 10^+03^	9.804 × 10^−01^	2.484 × 10^+03^	9.184 × 10^−03^
	6	2.418 × 10^+03^	9.068 × 10^−01^	2.438 × 10^+03^	6.645 × 10^−01^	2.462 × 10^+03^	7.520 × 10^−01^	2.486 × 10^+03^	9.283 × 10^−01^	2.489 × 10^+03^	4.272 × 10^−01^	2.582 × 10^+03^	6.756 × 10^−03^
	8	2.502 × 10^+03^	2.733 × 10^−01^	2.541 × 10^+03^	7.706 × 10^−01^	2.527 × 10^+03^	4.829 × 10^−01^	2.527 × 10^+03^	1.037 × 10^−01^	2.543 × 10^+03^	5.780 × 10^−01^	2.582 × 10^+03^	1.078 × 10^−03^
M7	2	1.830 × 10^+03^	9.584 × 10^−01^	1.878 × 10^+03^	9.725 × 10^−02^	1.884 × 10^+03^	4.415 × 10^−03^	1.867 × 10^+03^	9.208 × 10^−01^	1.823 × 10^+03^	2.722 × 10^−01^	1.991 × 10^+03^	4.926 × 10^−03^
	4	1.937 × 10^+03^	8.134 × 10^−01^	1.919 × 10^+03^	5.443 × 10^−01^	1.996 × 10^+03^	8.147 × 10^−01^	1.935 × 10^+03^	1.655 × 10^−01^	1.926 × 10^+03^	9.502 × 10^−01^	2.092 × 10^+03^	2.248 × 10^−03^
	6	2.086 × 10^+03^	7.735 × 10^−01^	2.031 × 10^+03^	3.954 × 10^−01^	2.031 × 10^+03^	1.736 × 10^−01^	2.064 × 10^+03^	5.458 × 10^−01^	2.012 × 10^+03^	6.190 × 10^−01^	2.125 × 10^+03^	9.585 × 10^−03^
	8	2.122 × 10^+03^	3.275 × 10^−01^	2.119 × 10^+03^	2.701 × 10^−01^	2.129 × 10^+03^	1.146 × 10^−01^	2.151 × 10^+03^	6.068 × 10^−01^	2.136 × 10^+03^	7.348 × 10^−01^	2.150 × 10^+03^	3.670 × 10^−01^
M8	2	2.193 × 10^+03^	8.043 × 10^−01^	2.168 × 10^+03^	2.752 × 10^−01^	2.174 × 10^+03^	9.430 × 10^−01^	2.246 × 10^+03^	9.186 × 10^−01^	2.256 × 10^+03^	1.558 × 10^−01^	2.381 × 10^+03^	6.996 × 10^−01^
	4	2.346 × 10^+03^	6.934 × 10^−01^	2.308 × 10^+03^	7.018 × 10^−01^	2.397 × 10^+03^	9.771 × 10^−02^	2.384 × 10^+03^	8.133 × 10^−01^	2.306 × 10^+03^	6.269 × 10^−01^	2.460 × 10^+03^	3.807 × 10^−03^
	6	2.429 × 10^+03^	2.730 × 10^−01^	2.411 × 10^+03^	6.778 × 10^−01^	2.403 × 10^+03^	4.274 × 10^−01^	2.439 × 10^+03^	7.378 × 10^−01^	2.466 × 10^+03^	2.616 × 10^−01^	2.577 × 10^+03^	2.923 × 10^−03^
	8	2.516 × 10^+03^	4.396 × 10^−01^	2.522 × 10^+03^	8.564 × 10^−01^	2.527 × 10^+03^	6.899 × 10^−01^	2.543 × 10^+03^	3.740 × 10^−01^	2.548 × 10^+03^	8.250 × 10^−01^	2.578 × 10^+03^	2.502 × 10^−04^
M9	2	1.899 × 10^+03^	6.740 × 10^−01^	1.720 × 10^+03^	4.882 × 10^−01^	1.803 × 10^+03^	5.393 × 10^−01^	1.751 × 10^+03^	5.164 × 10^−01^	1.703 × 10^+03^	2.144 × 10^−01^	1.990 × 10^+03^	1.143 × 10^−03^
	4	1.936 × 10^+03^	2.621 × 10^−01^	1.918 × 10^+03^	8.270 × 10^−01^	1.938 × 10^+03^	2.595 × 10^−01^	1.928 × 10^+03^	3.427 × 10^−01^	1.992 × 10^+03^	7.983 × 10^−01^	2.072 × 10^+03^	9.555 × 10^−03^
	6	2.080 × 10^+03^	4.779 × 10^−01^	2.091 × 10^+03^	5.835 × 10^−01^	2.091 × 10^+03^	3.184 × 10^−01^	2.076 × 10^+03^	2.208 × 10^−01^	2.044 × 10^+03^	6.504 × 10^−01^	2.116 × 10^+03^	7.134 × 10^−03^
	8	2.188 × 10^+03^	3.904 × 10^−01^	2.110 × 10^+03^	1.766 × 10^−01^	2.148 × 10^+03^	6.578 × 10^−01^	2.117 × 10^+03^	3.469 × 10^−01^	2.109 × 10^+03^	8.407 × 10^−01^	2.293 × 10^+03^	7.798 × 10^−03^
M10	2	1.719 × 10^+03^	1.438 × 10^−02^	1.743 × 10^+03^	7.830 × 10^−01^	1.884 × 10^+03^	6.177 × 10^−01^	1.895 × 10^+03^	6.832 × 10^−01^	1.870 × 10^+03^	7.778 × 10^−02^	1.969 × 10^+03^	6.079 × 10^−03^
	4	1.909 × 10^+03^	3.621 × 10^−01^	1.910 × 10^+03^	4.601 × 10^−01^	1.912 × 10^+03^	4.820 × 10^−01^	1.956 × 10^+03^	4.957 × 10^−01^	1.916 × 10^+03^	2.702 × 10^−01^	2.051 × 10^+03^	6.521 × 10^−03^
	6	2.081 × 10^+03^	1.636 × 10^−01^	2.054 × 10^+03^	8.964 × 10^−01^	2.037 × 10^+03^	1.358 × 10^−01^	2.008 × 10^+03^	5.350 × 10^−01^	2.066 × 10^+03^	2.550 × 10^−01^	2.154 × 10^+03^	3.527 × 10^−03^
	8	2.125 × 10^+03^	4.139 × 10^−01^	2.121 × 10^+03^	4.987 × 10^−01^	2.170 × 10^+03^	2.339 × 10^−01^	2.162 × 10^+03^	3.894 × 10^−02^	2.132 × 10^+03^	4.937 × 10^−01^	2.249 × 10^+03^	1.102 × 10^−03^
M11	2	1.787 × 10^+03^	5.036 × 10^−01^	1.848 × 10^+03^	2.007 × 10^−01^	1.750 × 10^+03^	8.128 × 10^−03^	1.817 × 10^+03^	1.156 × 10^−01^	1.845 × 10^+03^	7.805 × 10^−01^	1.973 × 10^+03^	3.508 × 10^−03^
	4	1.988 × 10^+03^	7.509 × 10^−01^	1.998 × 10^+03^	8.576 × 10^−02^	1.963 × 10^+03^	4.031 × 10^−01^	1.928 × 10^+03^	2.261 × 10^−01^	1.932 × 10^+03^	6.414 × 10^−01^	2.048 × 10^+03^	4.671 × 10^−01^
	6	2.077 × 10^+03^	7.419 × 10^−02^	2.068 × 10^+03^	1.906 × 10^−01^	2.064 × 10^+03^	3.102 × 10^−01^	2.025 × 10^+03^	2.401 × 10^−02^	2.036 × 10^+03^	2.432 × 10^−01^	2.182 × 10^+03^	9.253 × 10^−01^
	8	2.165 × 10^+03^	8.111 × 10^−01^	2.150 × 10^+03^	1.194 × 10^−01^	2.168 × 10^+03^	1.836 × 10^−01^	2.116 × 10^+03^	3.910 × 10^−01^	2.156 × 10^+03^	1.398 × 10^−01^	2.228 × 10^+03^	8.540 × 10^−01^
M12	2	1.923 × 10^+03^	6.196 × 10^−01^	2.098 × 10^+03^	4.279 × 10^−01^	1.925 × 10^+03^	7.119 × 10^−01^	2.085 × 10^+03^	3.927 × 10^−01^	2.060 × 10^+03^	7.114 × 10^−02^	2.169 × 10^+03^	3.564 × 10^−03^
	4	2.152 × 10^+03^	5.787 × 10^−01^	2.142 × 10^+03^	9.525 × 10^−01^	2.164 × 10^+03^	3.607 × 10^−01^	2.131 × 10^+03^	3.459 × 10^−01^	2.132 × 10^+03^	5.931 × 10^−01^	2.277 × 10^+03^	3.058 × 10^−03^
	6	2.284 × 10^+03^	2.190 × 10^−02^	2.233 × 10^+03^	6.926 × 10^−01^	2.275 × 10^+03^	3.104 × 10^−01^	2.295 × 10^+03^	9.227 × 10^−01^	2.209 × 10^+03^	3.474 × 10^−01^	2.331 × 10^+03^	4.266 × 10^−03^
	8	2.363 × 10^+03^	1.109 × 10^−01^	2.367 × 10^+03^	1.743 × 10^−01^	2.337 × 10^+03^	4.451 × 10^−01^	2.332 × 10^+03^	5.746 × 10^−01^	2.330 × 10^+03^	5.481 × 10^−01^	2.414 × 10^+03^	2.103 × 10^−03^
Friedman		4.15		4.15		3.81		3.85		3.94		1.10	
Final Rank		5		5		2		3		4		1	

**Table 4:** Wilcoxon rank sum test results.

Fun	nTH	PRO	HEOA	PO	QHDBO	IMODE
M1	2	1.3642 × 10^−10^/−	1.9289 × 10^−10^/−	5.0678 × 10^−10^/−	3.1608 × 10^−10^/−	1.4736 × 10^−10^/−
	4	5.8498 × 10^−04^/−	9.9219 × 10^−10^/−	6.5623 × 10^−10^/−	9.7275 × 10^−10^/−	4.5822 × 10^−10^/−
	6	2.0318 × 10^−05^/−	5.0506 × 10^−06^/−	6.2763 × 10^−06^/−	2.6400 × 10^−06^/−	8.6537 × 10^−10^/−
	8	4.9936 × 10^−06^/−	7.4739 × 10^−06^/−	5.2109 × 10^−10^/−	4.9712 × 10^−10^/−	2.7877 × 10^−10^/−
M2	2	6.4912 × 10^−05^/−	4.4042 × 10^−06^/−	1.6101 × 10^−10^/−	7.7586 × 10^−10^/−	5.8618 × 10^−10^/−
	4	5.5287 × 10^−10^/−	7.9693 × 10^−06^/−	2.5759 × 10^−05^/−	8.4656 × 10^−05^/−	6.6049 × 10^−10^/−
	6	1.1824 × 10^−10^/−	5.2306 × 10^−06^/−	2.1946 × 10^−05^/−	5.0859 × 10^−05^/−	9.1871 × 10^−08^/−
	8	3.3066 × 10^−10^/−	3.7303 × 10^−06^/−	2.4485 × 10^−05^/−	6.7861 × 10^−05^/−	4.7468 × 10^−07^/−
M3	2	6.5368 × 10^−04^/−	3.4066 × 10^−05^/−	5.5765 × 10^−05^/−	2.0654 × 10^−04^/−	2.1267 × 10^−07^/−
	4	1.5440 × 10^−04^/−	1.6304 × 10^−07^/−	4.6252 × 10^−05^/−	8.6085 × 10^−04^/−	9.9296 × 10^−07^/−
	6	1.7762 × 10^−04^/−	6.0955 × 10^−05^/−	7.8324 × 10^−05^/−	1.6428 × 10^−04^/−	7.1721 × 10^−08^/−
	8	3.8749 × 10^−04^/−	3.0658 × 10^−05^/−	7.4577 × 10^−05^/−	7.7117 × 10^−04^/−	2.8969 × 10^−07^/−
M4	2	4.6158 × 10^−04^/−	3.3361 × 10^−05^/−	6.6339 × 10^−05^/−	3.5820 × 10^−04^/−	6.7440 × 10^−07^/−
	4	8.6778 × 10^−04^/−	4.1070 × 10^−05^/−	8.4311 × 10^−10^/−	7.9695 × 10^−05^/−	5.6552 × 10^−07^/−
	6	6.3869 × 10^−04^/−	1.1849 × 10^−05^/−	8.9667 × 10^−10^/−	2.5410 × 10^−05^/−	1.8045 × 10^−07^/−
	8	3.2806 × 10^−04^/−	4.0863 × 10^−05^/−	7.1984 × 10^−10^/−	7.5516 × 10^−05^/−	6.2633 × 10^−07^/−
M5	2	1.0676 × 10^−04^/−	7.1504 × 10^−05^/−	7.0213 × 10^−10^/−	5.4913 × 10^−06^/−	1.6192 × 10^−07^/−
	4	4.5783 × 10^−04^/+	8.6690 × 10^−05^/−	2.2766 × 10^−10^/−	1.9205 × 10^−05^/−	5.6007 × 10^−10^/−
	6	8.0291 × 10^−04^/−	3.3926 × 10^−05^/−	7.4347 × 10^−10^/−	9.6063 × 10^−06^/−	1.8141 × 10^−10^/−
	8	9.0346 × 10^−04^/−	2.4084 × 10^−10^/−	5.1583 × 10^−10^/−	5.1951 × 10^−05^/−	9.5299 × 10^−10^/−
M6	2	1.9730 × 10^−10^/−	6.7358 × 10^−10^/−	7.3108 × 10^−10^/−	8.3355 × 10^−10^/−	5.3721 × 10^−10^/−
	4	7.3229 × 10^−08^/−	6.2939 × 10^−10^/−	9.3810 × 10^−10^/−	2.9181 × 10^−10^/−	5.3625 × 10^−10^/−
	6	2.0756 × 10^−10^/−	8.3014 × 10^−10^/−	9.7863 × 10^−10^/−	4.1487 × 10^−05^/−	7.7428 × 10^−10^/−
	8	7.5736 × 10^−10^/−	8.6631 × 10^−10^/−	4.7379 × 10^−10^/−	6.5172 × 10^−05^/−	1.8356 × 10^−10^/−
M7	2	5.5886 × 10^−10^/−	3.1267 × 10^−10^/−	3.5775 × 10^−08^/−	3.5684 × 10^−05^/−	3.1033 × 10^−10^
	4	6.5454 × 10^−06^/−	3.1429 × 10^−10^/−	3.0008 × 10^−07^/−	3.9868 × 10^−05^/−	2.6549 × 10^−05^/−
	6	5.8425 × 10^−06^/−	9.1127 × 10^−10^/−	8.8427 × 10^−07^/−	8.7889 × 10^−05^/−	6.2331 × 10^−07^/−
	8	8.0178 × 10^−06^/−	5.7071 × 10^−10^/−	3.7543 × 10^−07^/−	9.2456 × 10^−05^/+	3.5564 × 10^−05^/−
M8	2	2.7575 × 10^−06^/−	5.4833 × 10^−10^/−	7.3774 × 10^−07^/−	2.0791 × 10^−05^/−	1.0050 × 10^−05^/−
	4	2.8384 × 10^−06^/−	2.7683 × 10^−10^/−	1.8976 × 10^−07^/−	9.0112 × 10^−05^/−	8.3328 × 10^−05^/−
	6	4.5143 × 10^−06^/−	9.6078 × 10^−10^/−	7.7697 × 10^−07^/−	7.2491 × 10^−05^/−	8.4497 × 10^−05^/−
	8	8.2140 × 10^−06^/−	7.4315 × 10^−10^/−	3.3812 × 10^−08^/−	2.1328 × 10^−05^/−	4.3536 × 10^−05^/−
M9	2	5.8162 × 10^−06^/−	1.4464 × 10^−10^/−	4.1826 × 10^−07^/−	4.6928 × 10^−05^/−	1.7966 × 10^−05^/−
	4	6.6693 × 10^−06^/−	9.2293 × 10^−10^/−	9.3306 × 10^−07^/−	9.2167 × 10^−05^/−	3.5767 × 10^−05^/−
	6	7.5149 × 10^−06^/−	5.8577 × 10^−10^/−	1.7713 × 10^−10^/−	1.1862 × 10^−05^/−	6.5782 × 10^−05^/−
	8	8.7296 × 10^−06^/−	3.8752 × 10^−10^/−	6.5129 × 10^−10^/−	7.0887 × 10^−05^/−	9.5371 × 10^−05^/−
M10	2	7.1784 × 10^−06^/−	9.0082 × 10^−10^/−	7.6108 × 10^−10^/−	5.3151 × 10^−05^/−	2.5902 × 10^−05^/−
	4	4.3945 × 10^−06^/−	2.1765 × 10^−10^/−	2.7176 × 10^−10^/−	1.8635 × 10^−04^/−	7.7174 × 10^−10^/−
	6	9.0192 × 10^−10^/−	4.7446 × 10^−10^/−	1.6493 × 10^−10^/−	5.5588 × 10^−04^/−	4.8579 × 10^−10^/−
	8	2.4785 × 10^−05^/−	8.3432 × 10^−10^/−	5.4173 × 10^−06^/−	8.6634 × 10^−04^/−	7.9227 × 10^−05^/−
M11	2	1.6496 × 10^−05^/−	6.7877 × 10^−10^/−	4.7899 × 10^−07^/−	2.4490 × 10^−04^/−	9.8824 × 10^−05^/−
	4	5.7133 × 10^−05^/−	2.4253 × 10^−10^/−	3.2596 × 10^−07^/−	2.6633 × 10^−04^/−	3.2522 × 10^−05^/−
	6	3.7551 × 10^−05^/−	9.8707 × 10^−10^/−	8.6215 × 10^−08^/−	4.4853 × 10^−04^/−	8.3948 × 10^−05^/−
	8	2.8655 × 10^−05^/−	3.4994 × 10^−10^/−	3.2291 × 10^−07^/−	1.0395 × 10^−05^/−	5.1242 × 10^−05^/−
M12	2	5.0533 × 10^−05^/−	5.9923 × 10^−10^/−	7.5117 × 10^−07^/−	9.5975 × 10^−04^/−	2.0433 × 10^−05^/−
	4	1.4586 × 10^−05^/−	7.3389 × 10^−10^/−	2.5219 × 10^−10^/−	7.1579 × 10^−04^/−	4.2362 × 10^−05^/−
	6	1.9028 × 10^−10^/−	7.9507 × 10^−10^/−	1.8715 × 10^−10^/−	4.1668 × 10^−04^/−	4.3946 × 10^−05^/−
	8	8.1143 × 10^−10^/−	6.4769 × 10^−10^/−	3.2535 × 10^−10^/−	1.2204 × 10^−10^/−	8.4073 × 10^−10^/−
^+/−/=^	NA	1/47/0	0/48/0	0/48/0	1/47/0	0/48/0

**Table 5:** PSNR values.

Fun	nTH	PRO		HEOA		PO		QHDBO		IMODE		ANBPO	
		Mean	Std	Mean	Std	Mean	Std	Mean	Std	Mean	Std	Mean	Std
M1	2	18.948	6.431 × 10^−03^	18.871	5.434 × 10^−03^	18.179	7.396 × 10^−03^	18.282	6.507 × 10^−03^	18.996	6.192 × 10^−03^	19.737	7.942 × 10^−03^
	4	23.029	2.215 × 10^−03^	23.509	3.781 × 10^−03^	23.710	5.963 × 10^−03^	23.673	9.685 × 10^−03^	23.415	8.716 × 10^−03^	24.061	5.242 × 10^−03^
	6	25.110	9.285 × 10^−03^	25.376	2.059 × 10^−03^	25.186	3.874 × 10^−03^	25.984	9.170 × 10^−03^	25.823	2.748 × 10^−03^	26.669	2.993 × 10^−03^
	8	26.198	5.062 × 10^−03^	26.106	4.016 × 10^−03^	26.490	1.290 × 10^−03^	26.826	8.936 × 10^−03^	26.983	6.533 × 10^−03^	27.392	1.871 × 10^−03^
M2	2	18.398	4.722 × 10^−03^	18.960	3.664 × 10^−03^	18.599	8.896 × 10^−03^	18.935	9.255 × 10^−03^	18.420	7.506 × 10^−03^	19.081	2.716 × 10^−03^
	4	23.749	8.245 × 10^−03^	23.598	7.703 × 10^−03^	23.758	8.988 × 10^−03^	23.296	3.130 × 10^−03^	23.490	5.546 × 10^−03^	24.643	7.931 × 10^−03^
	6	25.790	3.760 × 10^−03^	25.708	6.644 × 10^−03^	25.577	2.420 × 10^−03^	25.946	3.913 × 10^−03^	25.100	1.942 × 10^−03^	26.924	9.338 × 10^−03^
	8	27.451	8.446 × 10^−03^	27.070	7.790 × 10^−03^	27.934	1.613 × 10^−03^	27.579	7.811 × 10^−03^	27.825	1.000 × 10^−03^	28.566	2.770 × 10^−03^
M3	2	18.761	4.736 × 10^−03^	18.914	6.974 × 10^−03^	18.052	3.960 × 10^−03^	18.747	3.189 × 10^−03^	18.356	1.548 × 10^−03^	19.087	2.699 × 10^−03^
	4	23.526	6.419 × 10^−03^	23.425	7.065 × 10^−03^	23.006	1.769 × 10^−03^	23.814	7.351 × 10^−03^	23.368	9.427 × 10^−03^	24.296	4.209 × 10^−03^
	6	25.915	4.281 × 10^−03^	25.183	1.710 × 10^−03^	25.164	7.487 × 10^−03^	25.756	3.281 × 10^−03^	25.207	4.932 × 10^−03^	26.759	3.714 × 10^−03^
	8	27.186	1.770 × 10^−03^	27.785	3.982 × 10^−03^	27.593	5.922 × 10^−03^	27.703	9.438 × 10^−03^	27.024	8.584 × 10^−03^	28.880	4.234 × 10^−03^
M4	2	19.764	2.542 × 10^−03^	19.613	4.726 × 10^−03^	19.556	8.408 × 10^−03^	19.807	3.400 × 10^−03^	19.130	4.268 × 10^−03^	21.424	4.715 × 10^−03^
	4	24.570	4.402 × 10^−03^	24.055	9.940 × 10^−03^	24.490	5.727 × 10^−03^	24.907	8.926 × 10^−03^	24.749	7.706 × 10^−03^	26.248	5.814 × 10^−03^
	6	26.671	7.628 × 10^−03^	26.966	7.511 × 10^−03^	26.796	7.843 × 10^−03^	26.243	7.350 × 10^−03^	26.055	4.836 × 10^−03^	28.147	9.914 × 10^−03^
	8	27.800	4.984 × 10^−03^	27.724	5.930 × 10^−03^	27.502	5.802 × 10^−03^	27.528	4.162 × 10^−03^	27.860	5.981 × 10^−03^	29.476	5.049 × 10^−03^
M5	2	19.696	9.531 × 10^−03^	19.525	3.142 × 10^−03^	19.294	8.580 × 10^−03^	19.866	1.327 × 10^−03^	19.808	2.690 × 10^−03^	20.164	5.219 × 10^−03^
	4	24.977	2.613 × 10^−03^	24.671	4.560 × 10^−03^	24.513	8.682 × 10^−03^	24.440	7.126 × 10^−03^	24.185	1.014 × 10^−03^	24.377	3.418 × 10^−03^
	6	26.838	6.689 × 10^−03^	26.061	3.680 × 10^−03^	26.462	2.890 × 10^−03^	26.342	1.909 × 10^−03^	26.821	4.198 × 10^−03^	28.122	1.387 × 10^−03^
	8	27.593	2.704 × 10^−03^	27.596	7.082 × 10^−03^	27.560	1.604 × 10^−03^	27.831	8.555 × 10^−03^	27.858	6.422 × 10^−03^	29.401	9.179 × 10^−03^
M6	2	16.463	1.833 × 10^−03^	16.178	2.184 × 10^−03^	16.459	7.405 × 10^−03^	16.772	3.672 × 10^−03^	16.048	2.002 × 10^−03^	17.416	1.503 × 10^−03^
	4	23.747	7.515 × 10^−03^	23.996	9.112 × 10^−03^	23.719	5.363 × 10^−03^	23.204	4.019 × 10^−03^	23.218	7.900 × 10^−03^	24.750	5.118 × 10^−03^
	6	25.732	1.062 × 10^−03^	25.329	1.404 × 10^−03^	25.101	1.534 × 10^−03^	25.446	2.963 × 10^−03^	25.439	7.117 × 10^−03^	26.017	6.295 × 10^−03^
	8	27.520	8.208 × 10^−03^	27.580	4.437 × 10^−03^	27.029	7.639 × 10^−03^	27.744	3.822 × 10^−03^	27.310	9.557 × 10^−03^	28.530	1.587 × 10^−03^
M7	2	16.420	1.003 × 10^−03^	16.253	5.090 × 10^−03^	16.519	9.744 × 10^−03^	16.255	4.468 × 10^−03^	16.121	5.017 × 10^−03^	17.795	5.527 × 10^−03^
	4	25.157	1.569 × 10^−03^	25.191	4.550 × 10^−03^	25.968	8.599 × 10^−03^	25.928	5.191 × 10^−03^	25.672	4.932 × 10^−03^	25.985	2.810 × 10^−03^
	6	26.741	7.496 × 10^−03^	26.948	1.184 × 10^−03^	26.358	8.283 × 10^−03^	26.736	2.786 × 10^−03^	26.396	9.040 × 10^−03^	27.502	9.672 × 10^−03^
	8	27.314	3.608 × 10^−03^	27.745	5.075 × 10^−03^	27.564	4.140 × 10^−03^	27.758	8.071 × 10^−03^	27.109	2.013 × 10^−03^	27.451	3.811 × 10^−03^
M8	2	19.270	6.595 × 10^−03^	19.196	6.562 × 10^−03^	19.417	8.707 × 10^−03^	19.634	1.081 × 10^−03^	19.154	6.760 × 10^−03^	20.661	3.827 × 10^−03^
	4	23.669	6.055 × 10^−03^	23.049	9.503 × 10^−03^	23.769	3.038 × 10^−03^	23.215	3.552 × 10^−03^	23.648	2.374 × 10^−03^	24.083	1.071 × 10^−03^
	6	25.961	4.647 × 10^−03^	25.874	2.147 × 10^−03^	25.437	9.950 × 10^−03^	25.824	4.299 × 10^−03^	25.246	3.990 × 10^−03^	26.069	9.972 × 10^−03^
	8	27.793	5.016 × 10^−03^	27.271	5.019 × 10^−03^	27.279	7.955 × 10^−03^	27.755	4.211 × 10^−03^	27.904	6.577 × 10^−03^	28.909	1.244 × 10^−03^
M9	2	18.239	6.615 × 10^−03^	18.390	5.847 × 10^−03^	18.144	1.292 × 10^−03^	18.154	4.918 × 10^−03^	18.578	8.464 × 10^−03^	19.475	3.923 × 10^−03^
	4	23.685	2.169 × 10^−03^	23.965	8.662 × 10^−03^	23.499	8.968 × 10^−03^	23.582	1.862 × 10^−03^	23.266	2.991 × 10^−03^	24.987	2.964 × 10^−03^
	6	25.158	7.461 × 10^−03^	25.592	7.900 × 10^−03^	25.014	2.560 × 10^−03^	25.734	3.023 × 10^−03^	25.537	8.125 × 10^−03^	26.813	6.944 × 10^−03^
	8	26.661	6.750 × 10^−03^	26.199	9.208 × 10^−03^	26.876	7.285 × 10^−03^	26.624	6.151 × 10^−03^	26.574	4.887 × 10^−03^	27.656	5.911 × 10^−03^
M10	2	18.880	4.788 × 10^−03^	18.430	5.841 × 10^−03^	18.753	7.874 × 10^−03^	18.051	7.757 × 10^−03^	18.789	1.816 × 10^−03^	19.566	1.263 × 10^−03^
	4	23.831	6.837 × 10^−03^	23.802	5.846 × 10^−03^	23.355	5.962 × 10^−03^	23.966	7.022 × 10^−03^	23.268	2.739 × 10^−03^	24.146	7.982 × 10^−03^
	6	25.884	2.186 × 10^−03^	25.702	2.248 × 10^−03^	25.812	1.882 × 10^−03^	25.735	8.936 × 10^−03^	25.338	9.046 × 10^−03^	26.532	4.592 × 10^−03^
	8	27.423	1.888 × 10^−03^	27.891	7.120 × 10^−03^	27.854	4.631 × 10^−03^	27.743	4.522 × 10^−03^	27.964	5.321 × 10^−03^	28.334	7.484 × 10^−03^
M11	2	18.666	8.069 × 10^−03^	18.624	4.246 × 10^−03^	18.063	2.530 × 10^−03^	18.816	4.687 × 10^−03^	18.210	3.716 × 10^−03^	19.615	5.701 × 10^−03^
	4	23.492	2.167 × 10^−03^	23.333	3.818 × 10^−03^	23.981	4.918 × 10^−03^	23.020	5.781 × 10^−03^	23.367	5.340 × 10^−03^	24.994	3.561 × 10^−03^
	6	25.346	5.592 × 10^−03^	25.502	3.352 × 10^−03^	25.593	5.235 × 10^−03^	25.829	4.435 × 10^−03^	25.671	4.580 × 10^−03^	26.035	2.118 × 10^−03^
	8	27.458	6.636 × 10^−03^	27.048	2.853 × 10^−03^	27.250	4.665 × 10^−03^	27.044	9.680 × 10^−03^	27.800	1.171 × 10^−03^	28.530	2.115 × 10^−03^
M12	2	19.477	5.922 × 10^−03^	19.043	5.443 × 10^−03^	19.995	9.523 × 10^−03^	19.220	9.256 × 10^−03^	19.090	2.938 × 10^−03^	21.642	6.402 × 10^−03^
	4	24.152	6.922 × 10^−03^	24.981	8.576 × 10^−03^	24.824	9.627 × 10^−03^	24.792	4.818 × 10^−03^	24.723	6.732 × 10^−03^	26.757	7.012 × 10^−03^
	6	26.892	6.377 × 10^−03^	26.936	4.513 × 10^−03^	26.493	9.232 × 10^−03^	26.586	1.399 × 10^−03^	26.785	4.522 × 10^−03^	28.944	1.292 × 10^−03^
	8	27.645	2.737 × 10^−03^	27.478	1.118 × 10^−03^	27.275	4.327 × 10^−03^	27.815	5.651 × 10^−03^	27.362	8.295 × 10^−03^	29.324	7.322 × 10^−03^
Friedman		3.65		4.02		4.29		3.56		4.33		1.15	
Final Rank		3		4		5		2		6		1	

**Table 6:** SSIM values.

Fun	nTH	PRO		HEOA		PO		QHDBO		IMODE		ANBPO	
		Mean	Std	Mean	Std	Mean	Std	Mean	Std	Mean	Std	Mean	Std
M1	2	0.752	2.643 × 10^−03^	0.756	6.077 × 10^−03^	0.752	3.976 × 10^−04^	0.751	8.020 × 10^−05^	0.754	1.180 × 10^−04^	0.778	1.588 × 10^−07^
	4	0.784	1.237 × 10^−03^	0.782	5.389 × 10^−03^	0.783	2.103 × 10^−03^	0.788	3.215 × 10^−05^	0.788	6.708 × 10^−04^	0.801	1.714 × 10^−07^
	6	0.822	7.748 × 10^−03^	0.820	6.726 × 10^−03^	0.829	6.552 × 10^−03^	0.822	7.201 × 10^−05^	0.826	9.211 × 10^−04^	0.844	1.706 × 10^−07^
	8	0.878	3.964 × 10^−03^	0.872	8.733 × 10^−03^	0.874	3.454 × 10^−03^	0.872	3.717 × 10^−05^	0.879	4.729 × 10^−04^	0.882	1.636 × 10^−07^
M2	2	0.788	9.816 × 10^−03^	0.781	9.051 × 10^−03^	0.784	5.497 × 10^−03^	0.783	7.158 × 10^−05^	0.788	5.055 × 10^−04^	0.802	1.648 × 10^−07^
	4	0.804	6.565 × 10^−03^	0.804	3.465 × 10^−03^	0.801	7.470 × 10^−03^	0.801	2.362 × 10^−06^	0.802	4.222 × 10^−04^	0.834	1.069 × 10^−07^
	6	0.822	2.464 × 10^−03^	0.827	8.876 × 10^−03^	0.820	6.397 × 10^−03^	0.827	6.781 × 10^−05^	0.823	9.681 × 10^−04^	0.858	1.718 × 10^−07^
	8	0.871	7.032 × 10^−03^	0.880	2.774 × 10^−03^	0.874	4.130 × 10^−03^	0.872	9.117 × 10^−05^	0.875	7.286 × 10^−05^	0.895	1.422 × 10^−07^
M3	2	0.714	7.558 × 10^−03^	0.719	4.954 × 10^−03^	0.714	9.531 × 10^−03^	0.711	3.333 × 10^−05^	0.718	9.142 × 10^−04^	0.730	1.470 × 10^−07^
	4	0.759	1.682 × 10^−03^	0.754	8.266 × 10^−03^	0.758	2.875 × 10^−03^	0.751	8.104 × 10^−05^	0.750	9.519 × 10^−04^	0.783	1.839 × 10^−07^
	6	0.800	8.176 × 10^−03^	0.809	3.518 × 10^−04^	0.808	5.856 × 10^−03^	0.802	9.555 × 10^−05^	0.809	2.005 × 10^−04^	0.838	1.463 × 10^−07^
	8	0.855	1.303 × 10^−03^	0.859	5.973 × 10^−03^	0.853	9.529 × 10^−03^	0.857	1.264 × 10^−05^	0.850	2.556 × 10^−04^	0.874	1.323 × 10^−07^
M4	2	0.739	5.422 × 10^−03^	0.739	6.932 × 10^−03^	0.731	9.682 × 10^−04^	0.732	4.499 × 10^−05^	0.736	7.911 × 10^−04^	0.751	1.499 × 10^−07^
	4	0.782	9.262 × 10^−03^	0.780	4.459 × 10^−03^	0.789	8.671 × 10^−03^	0.787	2.758 × 10^−05^	0.789	9.956 × 10^−04^	0.801	1.613 × 10^−07^
	6	0.813	9.479 × 10^−03^	0.816	5.415 × 10^−03^	0.814	4.823 × 10^−03^	0.812	5.477 × 10^−05^	0.810	7.253 × 10^−04^	0.847	1.233 × 10^−07^
	8	0.859	7.569 × 10^−03^	0.857	4.458 × 10^−03^	0.852	9.781 × 10^−03^	0.855	9.194 × 10^−06^	0.860	8.859 × 10^−04^	0.885	1.395 × 10^−07^
M5	2	0.714	1.600 × 10^−04^	0.717	5.490 × 10^−03^	0.714	9.559 × 10^−03^	0.718	4.458 × 10^−05^	0.711	5.508 × 10^−04^	0.743	1.807 × 10^−07^
	4	0.787	1.346 × 10^−03^	0.788	5.936 × 10^−03^	0.788	4.118 × 10^−03^	0.788	5.605 × 10^−05^	0.783	4.025 × 10^−04^	0.783	1.563 × 10^−07^
	6	0.817	4.812 × 10^−03^	0.812	2.412 × 10^−03^	0.816	5.138 × 10^−03^	0.812	1.639 × 10^−05^	0.817	1.855 × 10^−04^	0.860	1.412 × 10^−07^
	8	0.863	7.429 × 10^−03^	0.869	7.705 × 10^−03^	0.864	6.743 × 10^−03^	0.865	6.225 × 10^−05^	0.870	7.001 × 10^−04^	0.882	1.527 × 10^−07^
M6	2	0.696	8.681 × 10^−03^	0.698	9.945 × 10^−03^	0.700	7.385 × 10^−03^	0.697	9.679 × 10^−05^	0.691	8.415 × 10^−04^	0.728	1.071 × 10^−07^
	4	0.759	4.026 × 10^−03^	0.753	5.587 × 10^−03^	0.751	1.084 × 10^−03^	0.752	4.436 × 10^−05^	0.756	5.586 × 10^−04^	0.763	1.571 × 10^−07^
	6	0.806	3.782 × 10^−03^	0.809	8.803 × 10^−03^	0.808	4.708 × 10^−04^	0.802	5.244 × 10^−05^	0.801	2.218 × 10^−04^	0.815	1.268 × 10^−07^
	8	0.851	9.798 × 10^−03^	0.859	1.112 × 10^−03^	0.856	7.189 × 10^−03^	0.859	1.745 × 10^−05^	0.857	6.549 × 10^−04^	0.863	1.117 × 10^−07^
M7	2	0.782	7.635 × 10^−03^	0.789	7.779 × 10^−04^	0.782	8.831 × 10^−03^	0.787	2.032 × 10^−05^	0.784	8.895 × 10^−04^	0.811	1.374 × 10^−07^
	4	0.824	8.008 × 10^−03^	0.830	6.911 × 10^−03^	0.824	5.685 × 10^−03^	0.821	6.014 × 10^−05^	0.823	5.882 × 10^−04^	0.855	1.602 × 10^−07^
	6	0.851	3.468 × 10^−03^	0.852	3.604 × 10^−03^	0.851	2.190 × 10^−03^	0.855	8.776 × 10^−05^	0.851	9.348 × 10^−05^	0.884	1.372 × 10^−07^
	8	0.882	4.518 × 10^−03^	0.884	5.880 × 10^−03^	0.889	1.814 × 10^−03^	0.882	9.813 × 10^−05^	0.883	7.566 × 10^−04^	0.887	1.580 × 10^−07^
M8	2	0.798	8.703 × 10^−03^	0.792	5.266 × 10^−03^	0.796	5.415 × 10^−03^	0.796	2.599 × 10^−05^	0.797	9.361 × 10^−05^	0.814	1.176 × 10^−07^
	4	0.825	9.483 × 10^−03^	0.821	4.083 × 10^−03^	0.828	5.120 × 10^−03^	0.828	9.692 × 10^−05^	0.826	8.452 × 10^−04^	0.847	1.526 × 10^−07^
	6	0.874	2.405 × 10^−03^	0.877	6.367 × 10^−03^	0.880	4.223 × 10^−03^	0.878	9.456 × 10^−05^	0.879	2.382 × 10^−04^	0.889	1.010 × 10^−07^
	8	0.899	5.310 × 10^−03^	0.893	5.681 × 10^−03^	0.894	7.969 × 10^−03^	0.898	4.193 × 10^−05^	0.892	9.386 × 10^−04^	0.907	1.189 × 10^−07^
M9	2	0.756	8.144 × 10^−03^	0.754	6.554 × 10^−03^	0.757	2.265 × 10^−04^	0.752	8.115 × 10^−05^	0.756	6.495 × 10^−04^	0.777	1.255 × 10^−07^
	4	0.782	2.582 × 10^−03^	0.785	9.374 × 10^−03^	0.787	4.008 × 10^−03^	0.781	2.158 × 10^−05^	0.781	1.551 × 10^−04^	0.805	1.265 × 10^−07^
	6	0.820	8.710 × 10^−03^	0.824	6.838 × 10^−03^	0.828	3.086 × 10^−03^	0.822	3.669 × 10^−05^	0.826	9.403 × 10^−04^	0.844	1.407 × 10^−07^
	8	0.874	1.014 × 10^−03^	0.873	1.129 × 10^−03^	0.879	5.269 × 10^−03^	0.878	1.959 × 10^−05^	0.875	3.415 × 10^−04^	0.889	1.035 × 10^−07^
M10	2	0.787	5.769 × 10^−03^	0.787	4.003 × 10^−03^	0.786	5.706 × 10^−03^	0.784	7.099 × 10^−05^	0.780	7.754 × 10^−04^	0.796	1.661 × 10^−07^
	4	0.808	9.965 × 10^−03^	0.806	9.339 × 10^−04^	0.808	3.055 × 10^−03^	0.800	3.720 × 10^−05^	0.805	8.917 × 10^−04^	0.832	1.115 × 10^−07^
	6	0.821	3.505 × 10^−03^	0.821	4.432 × 10^−03^	0.827	7.472 × 10^−04^	0.823	4.702 × 10^−05^	0.821	4.791 × 10^−04^	0.855	1.879 × 10^−07^
	8	0.879	9.525 × 10^−03^	0.880	7.301 × 10^−04^	0.872	9.392 × 10^−03^	0.876	2.024 × 10^−05^	0.878	3.000 × 10^−06^	0.895	1.778 × 10^−07^
M11	2	0.712	1.516 × 10^−03^	0.719	1.697 × 10^−03^	0.710	3.505 × 10^−03^	0.716	1.506 × 10^−05^	0.711	6.611 × 10^−05^	0.731	1.670 × 10^−07^
	4	0.758	3.075 × 10^−03^	0.760	3.079 × 10^−03^	0.760	4.222 × 10^−03^	0.757	6.337 × 10^−05^	0.754	2.883 × 10^−04^	0.793	1.296 × 10^−07^
	6	0.801	9.692 × 10^−03^	0.808	9.521 × 10^−03^	0.809	9.285 × 10^−03^	0.800	1.703 × 10^−05^	0.805	6.038 × 10^−04^	0.838	1.538 × 10^−07^
	8	0.855	8.048 × 10^−03^	0.858	5.214 × 10^−03^	0.856	6.452 × 10^−04^	0.855	1.718 × 10^−06^	0.850	4.815 × 10^−04^	0.876	1.002 × 10^−07^
M12	2	0.734	8.004 × 10^−03^	0.734	6.843 × 10^−03^	0.737	4.436 × 10^−03^	0.736	4.736 × 10^−05^	0.730	8.503 × 10^−04^	0.754	1.204 × 10^−07^
	4	0.788	4.352 × 10^−03^	0.783	5.876 × 10^−03^	0.790	7.261 × 10^−03^	0.788	3.007 × 10^−05^	0.788	6.040 × 10^−04^	0.806	1.892 × 10^−07^
	6	0.817	8.133 × 10^−03^	0.815	5.369 × 10^−04^	0.814	4.129 × 10^−03^	0.811	9.668 × 10^−05^	0.820	7.695 × 10^−04^	0.849	1.184 × 10^−07^
	8	0.859	4.379 × 10^−03^	0.856	8.538 × 10^−03^	0.859	8.583 × 10^−03^	0.854	2.483 × 10^−05^	0.856	6.861 × 10^−04^	0.889	1.203 × 10^−07^
Friedman		4.06		3.71		3.60		4.38		4.15		1.10	
Final Rank		4		3		2		6		5		1	

**Table 7:** FSIM values.

Fun	nTH	PRO		HEOA		PO		QHDBO		IMODE		ANBPO	
		Mean	Std	Mean	Std	Mean	Std	Mean	Std	Mean	Std	Mean	Std
M1	2	0.798	7.671 × 10^−10^	0.798	1.427 × 10^−10^	0.799	2.433 × 10^−10^	0.800	8.985 × 10^−10^	0.793	2.913 × 10^−10^	**0.801**	9.544 × 10^−10^
	4	0.825	2.919 × 10^−10^	0.822	6.869 × 10^−10^	0.827	4.706 × 10^−10^	0.827	1.268 × 10^−10^	0.828	1.297 × 10^−10^	**0.831**	1.410 × 10^−10^
	6	0.852	5.967 × 10^−10^	0.860	1.121 × 10^−10^	0.856	5.529 × 10^−10^	0.853	2.067 × 10^−10^	0.859	8.796 × 10^−10^	**0.863**	6.392 × 10^−10^
	8	0.878	3.223 × 10^−10^	0.873	8.520 × 10^−10^	0.870	1.153 × 10^−10^	0.872	1.914 × 10^−10^	0.879	7.789 × 10^−10^	**0.881**	5.637 × 10^−10^
M2	2	0.794	2.639 × 10^−10^	0.796	4.973 × 10^−10^	0.790	8.604 × 10^−10^	0.793	6.794 × 10^−10^	0.796	6.055 × 10^−10^	**0.805**	4.785 × 10^−10^
	4	0.820	8.796 × 10^−10^	0.824	2.833 × 10^−10^	0.824	2.461 × 10^−10^	0.830	3.970 × 10^−10^	0.826	6.208 × 10^−10^	**0.842**	9.479 × 10^−10^
	6	0.856	5.793 × 10^−10^	0.859	1.674 × 10^−10^	0.854	7.757 × 10^−10^	0.855	1.905 × 10^−10^	0.859	4.073 × 10^−10^	**0.873**	7.561 × 10^−10^
	8	0.874	5.700 × 10^−10^	0.880	1.008 × 10^−10^	0.873	7.701 × 10^−10^	0.880	3.857 × 10^−10^	0.877	3.013 × 10^−10^	**0.897**	2.136 × 10^−10^
M3	2	0.801	2.604 × 10^−10^	0.803	8.470 × 10^−10^	0.808	4.405 × 10^−10^	0.803	6.634 × 10^−10^	0.809	3.888 × 10^−10^	**0.817**	6.414 × 10^−10^
	4	0.831	5.514 × 10^−10^	0.834	2.320 × 10^−10^	0.832	9.171 × 10^−10^	0.839	3.347 × 10^−10^	0.832	7.324 × 10^−10^	**0.859**	6.433 × 10^−10^
	6	0.863	9.629 × 10^−10^	0.862	3.916 × 10^−10^	0.868	2.226 × 10^−10^	0.862	9.032 × 10^−10^	0.863	7.993 × 10^−10^	**0.882**	9.140 × 10^−10^
	8	0.876	3.382 × 10^−10^	0.872	3.255 × 10^−10^	0.877	4.873 × 10^−10^	0.876	8.729 × 10^−10^	0.878	2.738 × 10^−10^	**0.893**	6.079 × 10^−10^
M4	2	0.771	6.064 × 10^−10^	0.774	9.973 × 10^−10^	0.780	1.845 × 10^−10^	0.771	3.158 × 10^−10^	0.778	1.177 × 10^−10^	**0.802**	5.641 × 10^−10^
	4	0.804	8.604 × 10^−10^	0.800	2.854 × 10^−10^	0.801	3.434 × 10^−10^	0.801	4.915 × 10^−10^	0.802	8.067 × 10^−10^	**0.829**	5.578 × 10^−10^
	6	0.835	5.676 × 10^−10^	0.835	1.105 × 10^−10^	0.836	3.662 × 10^−10^	0.830	7.323 × 10^−10^	0.839	8.092 × 10^−10^	**0.859**	3.150 × 10^−10^
	8	0.858	2.787 × 10^−10^	0.857	6.825 × 10^−10^	0.859	3.111 × 10^−10^	0.860	4.983 × 10^−10^	0.854	8.341 × 10^−10^	**0.893**	9.650 × 10^−10^
M5	2	0.781	8.391 × 10^−10^	0.790	6.617 × 10^−10^	0.783	6.852 × 10^−10^	0.783	4.982 × 10^−10^	0.786	6.889 × 10^−10^	**0.800**	5.863 × 10^−10^
	4	0.813	1.329 × 10^−10^	0.817	5.602 × 10^−10^	**0.819**	3.138 × 10^−10^	**0.819**	1.645 × 10^−10^	0.816	6.383 × 10^−10^	0.818	4.627 × 10^−10^
	6	0.831	1.607 × 10^−10^	0.832	4.486 × 10^−10^	0.838	8.340 × 10^−10^	0.834	7.353 × 10^−10^	0.831	2.477 × 10^−10^	**0.874**	8.210 × 10^−10^
	8	0.854	9.556 × 10^−10^	0.857	2.336 × 10^−10^	0.856	4.751 × 10^−10^	0.852	7.427 × 10^−10^	0.859	1.125 × 10^−10^	**0.897**	3.600 × 10^−10^
M6	2	0.752	3.889 × 10^−10^	0.760	9.458 × 10^−10^	0.757	1.757 × 10^−10^	0.759	3.092 × 10^−10^	0.753	2.092 × 10^−10^	**0.773**	2.091 × 10^−10^
	4	0.795	3.121 × 10^−10^	0.796	5.610 × 10^−10^	0.796	1.543 × 10^−10^	0.792	4.536 × 10^−10^	0.792	4.435 × 10^−10^	**0.818**	9.195 × 10^−10^
	6	0.828	1.979 × 10^−10^	0.829	6.555 × 10^−10^	0.829	6.996 × 10^−10^	0.824	9.506 × 10^−10^	0.827	4.817 × 10^−10^	**0.842**	1.939 × 10^−10^
	8	0.855	9.146 × 10^−10^	0.853	9.433 × 10^−10^	0.855	6.145 × 10^−10^	0.860	5.015 × 10^−10^	0.860	6.151 × 10^−10^	**0.889**	4.784 × 10^−10^
M7	2	0.811	3.174 × 10^−10^	0.811	7.280 × 10^−10^	0.810	4.794 × 10^−10^	0.812	7.886 × 10^−10^	0.818	5.175 × 10^−10^	**0.821**	9.999 × 10^−10^
	4	0.839	1.771 × 10^−10^	0.832	1.306 × 10^−10^	0.836	2.525 × 10^−10^	0.831	5.607 × 10^−10^	0.837	1.601 × 10^−10^	**0.841**	8.062 × 10^−10^
	6	0.869	9.444 × 10^−10^	0.861	4.849 × 10^−10^	0.868	7.737 × 10^−10^	0.861	4.825 × 10^−10^	0.860	1.632 × 10^−10^	**0.872**	5.767 × 10^−10^
	8	0.876	5.568 × 10^−10^	0.872	9.277 × 10^−10^	0.870	4.472 × 10^−10^	0.875	1.156 × 10^−10^	**0.880**	3.822 × 10^−10^	0.878	9.195 × 10^−10^
M8	2	0.826	8.971 × 10^−10^	0.823	7.145 × 10^−10^	0.828	8.958 × 10^−10^	0.826	3.129 × 10^−10^	0.827	9.896 × 10^−10^	**0.834**	5.600 × 10^−10^
	4	0.849	1.531 × 10^−10^	0.842	6.552 × 10^−10^	0.844	5.370 × 10^−10^	0.846	4.742 × 10^−10^	0.846	6.971 × 10^−10^	**0.875**	4.977 × 10^−10^
	6	0.868	1.478 × 10^−10^	0.860	6.614 × 10^−10^	0.869	3.402 × 10^−10^	0.864	8.251 × 10^−10^	0.863	5.424 × 10^−10^	**0.883**	6.214 × 10^−10^
	8	0.891	7.119 × 10^−10^	0.893	8.060 × 10^−10^	0.892	6.556 × 10^−10^	0.891	3.667 × 10^−10^	0.891	4.384 × 10^−10^	**0.907**	5.019 × 10^−10^
M9	2	0.793	9.986 × 10^−10^	0.797	7.894 × 10^−10^	0.792	4.612 × 10^−10^	0.790	4.694 × 10^−10^	0.797	1.118 × 10^−10^	**0.801**	1.455 × 10^−10^
	4	0.821	7.033 × 10^−10^	0.821	6.354 × 10^−10^	0.823	8.126 × 10^−10^	0.825	3.741 × 10^−10^	0.822	8.662 × 10^−10^	**0.831**	1.137 × 10^−10^
	6	0.856	7.681 × 10^−10^	0.857	1.720 × 10^−10^	0.855	9.693 × 10^−10^	0.852	1.155 × 10^−10^	0.852	6.394 × 10^−10^	**0.865**	2.102 × 10^−10^
	8	0.872	1.839 × 10^−10^	0.879	1.873 × 10^−10^	0.870	9.710 × 10^−10^	0.879	5.005 × 10^−10^	0.879	3.250 × 10^−10^	**0.883**	5.175 × 10^−10^
M10	2	0.800	7.026 × 10^−10^	0.795	2.194 × 10^−10^	0.791	7.115 × 10^−10^	0.795	8.402 × 10^−10^	0.792	9.003 × 10^−10^	**0.802**	2.652 × 10^−10^
	4	0.824	3.238 × 10^−10^	0.825	1.209 × 10^−10^	0.830	3.773 × 10^−10^	0.826	2.237 × 10^−10^	0.829	1.837 × 10^−10^	**0.850**	4.144 × 10^−10^
	6	0.853	3.525 × 10^−10^	0.855	4.891 × 10^−10^	0.859	9.868 × 10^−10^	0.856	5.215 × 10^−10^	0.859	4.371 × 10^−10^	**0.875**	1.995 × 10^−10^
	8	0.879	9.666 × 10^−10^	0.878	8.274 × 10^−10^	0.876	1.575 × 10^−10^	0.874	5.380 × 10^−10^	0.872	6.101 × 10^−10^	**0.897**	1.206 × 10^−10^
M11	2	0.801	6.377 × 10^−10^	0.804	3.643 × 10^−10^	0.807	4.624 × 10^−10^	0.804	2.646 × 10^−10^	0.806	3.460 × 10^−10^	**0.810**	1.991 × 10^−10^
	4	0.833	2.882 × 10^−10^	0.836	8.051 × 10^−10^	0.836	5.154 × 10^−10^	0.837	2.010 × 10^−10^	0.839	2.509 × 10^−10^	**0.857**	3.079 × 10^−10^
	6	0.863	1.676 × 10^−10^	0.867	1.715 × 10^−10^	0.869	1.669 × 10^−10^	0.863	1.729 × 10^−10^	0.869	1.669 × 10^−10^	**0.889**	7.239 × 10^−10^
	8	0.872	7.825 × 10^−10^	0.871	4.413 × 10^−10^	0.873	2.579 × 10^−10^	0.870	8.019 × 10^−10^	0.871	6.703 × 10^−10^	**0.899**	5.731 × 10^−10^
M12	2	0.774	6.153 × 10^−10^	0.779	3.294 × 10^−10^	0.776	3.350 × 10^−10^	0.771	6.066 × 10^−10^	0.777	8.168 × 10^−10^	**0.805**	4.239 × 10^−10^
	4	0.806	2.949 × 10^−10^	0.801	9.813 × 10^−10^	0.806	4.758 × 10^−10^	0.802	7.147 × 10^−10^	0.800	6.686 × 10^−10^	**0.822**	1.175 × 10^−10^
	6	0.833	7.769 × 10^−10^	0.832	7.424 × 10^−10^	0.837	7.030 × 10^−10^	0.837	2.140 × 10^−10^	0.836	4.043 × 10^−10^	**0.851**	2.262 × 10^−10^
	8	0.856	3.042 × 10^−10^	0.855	8.737 × 10^−10^	0.858	6.157 × 10^−10^	0.851	6.032 × 10^−10^	0.860	1.685 × 10^−10^	**0.897**	4.434 × 10^−10^
Friedman		4.44		4.15		3.67		4.19		3.50		**1.06**	
Final Rank		6		4		3		5		2		**1**	

**Table 8:** Run time results.

Fun	nTH	PRO	HEOA	PO	QHDBO	IMODE	ANBPO
		Mean	Mean	Mean	Mean	Mean	Mean
M1	2	28.418	27.732	27.207	35.044	39.903	**15.872**
	4	24.512	28.908	33.846	36.770	39.979	**16.794**
	6	23.464	24.300	30.909	32.291	38.638	**19.305**
	8	22.784	**20.074**	26.257	35.075	34.071	21.193
M2	2	27.419	29.395	26.448	32.333	39.982	**19.741**
	4	25.149	27.124	34.035	35.442	35.360	**20.360**
	6	24.935	22.816	32.923	30.494	36.075	**20.240**
	8	26.584	21.500	30.867	36.214	37.853	**15.106**
M3	2	24.426	25.703	34.552	36.414	35.329	**19.073**
	4	25.513	24.394	28.710	31.650	38.250	**17.598**
	6	20.143	21.771	32.414	36.254	34.201	**17.671**
	8	**20.169**	29.250	28.480	32.327	34.022	21.456
M4	2	26.482	24.191	25.499	33.426	37.421	**17.294**
	4	23.438	21.553	34.666	30.731	35.716	**19.112**
	6	22.007	26.673	33.439	36.911	34.275	**15.070**
	8	24.364	23.343	33.860	35.526	39.444	**17.701**
M5	2	26.879	20.605	29.611	32.220	39.017	**18.892**
	4	27.305	26.592	29.953	32.992	33.499	**20.750**
	6	27.992	23.241	31.705	32.117	38.287	**17.666**
	8	24.145	21.654	26.104	31.038	36.422	**20.282**
M6	2	22.203	24.699	25.765	34.510	36.696	**21.163**
	4	28.377	26.593	26.356	30.962	36.618	**17.723**
	6	25.073	**20.860**	33.474	36.140	35.659	20.983
	8	22.162	22.441	34.902	35.936	36.514	**20.026**
M7	2	29.083	23.346	27.788	34.974	36.710	**19.411**
	4	23.179	23.147	30.020	34.872	38.901	**15.789**
	6	29.370	27.238	30.134	36.375	36.557	**19.072**
	8	26.347	28.823	29.418	35.208	39.064	**20.600**
M8	2	28.969	28.479	33.897	30.082	34.456	**19.406**
	4	28.659	27.214	33.084	30.700	33.066	**15.193**
	6	22.041	22.837	31.072	32.710	38.444	**17.133**
	8	24.313	29.987	33.631	31.249	37.243	**21.258**
M9	2	25.127	29.235	27.664	33.707	38.235	**18.514**
	4	20.308	23.832	27.896	32.329	35.982	**19.858**
	6	21.316	**20.951**	30.412	30.335	34.930	21.389
	8	26.414	**20.061**	29.903	31.622	39.414	20.975
M10	2	26.904	25.833	34.601	31.322	39.103	**16.341**
	4	26.366	29.611	33.333	34.433	38.777	**15.381**
	6	29.743	23.551	28.855	35.384	34.092	**19.018**
	8	21.983	29.155	32.242	32.647	35.574	**18.382**
M11	2	27.982	26.065	26.284	31.345	39.354	**18.038**
	4	21.640	21.216	26.877	34.835	37.051	**20.997**
	6	27.838	29.623	25.736	35.382	33.683	**17.983**
	8	20.425	24.790	25.219	30.543	35.112	**18.741**
M12	2	21.569	29.425	33.211	32.090	38.819	**20.157**
	4	22.223	26.895	28.659	31.042	35.850	**21.930**
	6	24.816	29.507	27.613	33.344	39.436	**20.267**
	8	29.805	20.535	25.413	34.566	34.154	**17.624**
Friedman		2.69	2.52	3.85	5.02	5.79	**1.13**
Final Rank		3	2	4	5	6	**1**

## Data Availability

If there is a reasonable need, you can request it from the corresponding author.
